# Analysis of a global wheat panel reveals a highly diverse introgression landscape and provides evidence for inter-homoeologue chromosomal recombination

**DOI:** 10.1007/s00122-024-04721-x

**Published:** 2024-09-28

**Authors:** Matthias Heuberger, Zoe Bernasconi, Mahmoud Said, Esther Jung, Gerhard Herren, Victoria Widrig, Hana Šimková, Beat Keller, Javier Sánchez-Martín, Thomas Wicker

**Affiliations:** 1 Department of Plant and Microbial Biology, University of Zurich, Zurich, Switzerland; 2 Centre of Plant Structural and Functional Genomics, Institute of Experimental Botany of the Czech Academy of Sciences, Olomouc, Czech Republic; 3 Agricultural Research Centre, Field Crops Research Institute, Giza, Egypt; 4 Department of Microbiology and Genetics, Spanish-Portuguese Agricultural Research Centre (CIALE), University of Salamanca, Salamanca, Spain

## Abstract

**Key message:**

This study highlights the agronomic potential of rare introgressions, as demonstrated by a major QTL for powdery mildew resistance on chromosome 7D. It further shows evidence for inter-homoeologue recombination in wheat.

**Abstract:**

Agriculturally important genes are often introgressed into crops from closely related donor species or landraces. The gene pool of hexaploid bread wheat (*Triticum aestivum)* is known to contain numerous such “alien” introgressions. Recently established high-quality reference genome sequences allow prediction of the size, frequency and identity of introgressed chromosome regions. Here, we characterise chromosomal introgressions in bread wheat using exome capture data from the WHEALBI collection. We identified 24,981 putative introgression segments of at least 2 Mb across 434 wheat accessions. Detailed study of the most frequent introgressions identified *T. timopheevii* or its close relatives as a frequent donor species. Importantly, 118 introgressions of at least 10 Mb were exclusive to single wheat accessions, revealing that large populations need to be studied to assess the total diversity of the wheat pangenome. In one case, a 14 Mb introgression in chromosome 7D, exclusive to cultivar Pamukale, was shown by QTL mapping to harbour a recessive powdery mildew resistance gene. We identified multiple events where distal chromosomal segments of one subgenome were duplicated in the genome and replaced the homoeologous segment in another subgenome. We propose that these examples are the results of inter-homoeologue recombination. Our study produced an extensive catalogue of the wheat introgression landscape, providing a resource for wheat breeding. Of note, the finding that the wheat gene pool contains numerous rare, but potentially important introgressions and chromosomal rearrangements has implications for future breeding.

**Supplementary Information:**

The online version contains supplementary material available at 10.1007/s00122-024-04721-x.

## Introduction

Bread wheat (*Triticum aestivum*) is a hexaploid species (genome formula AABBDD) that resulted from two allopolyploidisation events. The A, B and D subgenomes each have seven chromosomes referred to as homoeologues (Glover et al. 2016). The first polyploidisation occurred about 0.5 million years ago when *T. urartu* (AA) and a yet unknown donor, closely related to *Aegilops speltoides,* hybridised to form *T. turgidum* (AABB) (Marcussen et al. 2014). This tetraploid wheat, which began to be cultivated approximately 10,000 years ago in the Fertile Crescent, subsequently hybridised with *Ae. tauschii* to form bread wheat. Both these allopolyploidisation events acted as genetic bottlenecks, so bread wheat contains only a fraction of the genetic diversity found in the genomes of the three progenitor species. This reduced diversity is most pronounced in the D subgenome (Gaurav et al. 2021).

It is well known that wild relatives of crops and landraces are a rich resource of novel genes for exploitation in contemporary agriculture (Hajjar and Hodgkin 2007). The genetic diversity of this original gene pool was only partially incorporated into modern wheat varieties. One method to introduce genetic material from wild crop relatives is introgression breeding (Hao et al. 2020). Here, a crop variety is hybridised with a relative containing a desired trait. Genetic recombination between non-homologous chromosomes of wheat is suppressed mainly by the *Ph1* locus (Griffiths et al. 2006). Therefore, the *Ph1* locus is either removed before introgression breeding, or the cross is made with a species not containing the *Ph1* locus, such as *Ambylopyrum muticum* (King et al. 2017, 2019; Coombes et al. 2023). After a successful cross, the resulting offspring is subsequently backcrossed while selecting the offspring for the trait of interest. This is done to reduce potentially unwanted phenotypes originating from the donor genotype.

In bread wheat, introgression breeding has been widely used for many traits and genes of agronomic importance. Breeding for disease resistance has relied heavily on introgressions from wild or crop relatives. For example, the 1RS.1BL translocation where the short arm of wheat chromosome 1B is replaced by the short arm of rye (*Secale cereale*) chromosome 1R contains genes conferring resistance to four different fungal pathogens: *Lr26* for resistance to leaf rust (*Puccinia triticina*), *Yr9* for resistance to yellow rust (*P. striiformis*), *Sr31* for resistance to stem rust (*P. graminis*) and *Pm8* for resistance to powdery mildew (*Blumeria graminis* f. sp. *tritici*, or *Bgt*, Crespo-Herrera et al. 2017). Examples of other introgressed disease resistance genes are *Sr43* from *Thinopyrum elongatum* (Knott et al. 1977; Yu et al. 2023), *Lr9* (Wang et al. 2023), *Lr76*, *Yr70* (Bansal et al. 2020) from *Ae. umbellulata* and *Pm4b* from *T. carthlicum* (Sánchez-Martín et al. 2021).

Cytological analyses such as C-banding have been important tools to detect introgressions in specific genotypes, and they rely on differential staining of heterochromatin (Badaeva et al. 1991; Orlovskaya et al. 2020). A molecular method to determine the presence of introgressions is simple sequence repeat (SSR) analysis, where the differential amplification of SSR markers informs about the presence of alien chromatin (Orlovskaya et al. 2020). The advent of next generation sequencing (NGS) and the availability of reference wheat genomes now enable whole-genome analysis (Walkowiak et al. 2020). Genomic methods to identify introgressions include transposable element population analysis (Walkowiak et al. 2020; Wicker et al. 2022), k-mer-based approaches (https://github.com/Uauy-Lab/IBSpy, Ahmed et al. 2023) and single nucleotide polymorphism (SNP)-based methods (Cheng et al. 2019). Additionally, a straightforward way of introgression detection is the analysis of sequencing coverage depth (Keilwagen et al. 2019, 2022, 2023; Walkowiak et al. 2020; Kale et al. 2022). Here, genomic sequence reads (typically Illumina short reads) from a given wheat accession are mapped to a reference genome. In regions containing divergent haplotypes or introgressions, fewer sequence reads will map due to lower sequence homology. The method does not directly allow the differentiation between introgressions which are present in the reference genome and introgressions that are present in the sample. However, it is possible to overcome this limitation by using multiple genomes as reference.

To assess the current diversity of the modern wheat gene pool, several collections of wheat genotypes were assembled and made publicly available. In this study, we used the WHEALBI resource (Pont et al. 2019, www.whealbi.eu), a collection of 434 hexaploid genotypes which were categorised in the WHEALBI project into historic groups as landraces (deployment before 1935, *N* = 95), old cultivars (registered from 1936 to 1965, *N* = 99), cultivars (registered from 1966 to 1985, *N* = 130) and current varieties (registered after 1986, *N* = 99; 11 accessions are not categorised). By mapping publicly available exome capture reads from the WHEALBI collection onto the wheat reference genome (cv. Chinese Spring v2.1, Zhu et al. 2021), we identified a total of 24,981 putative introgressions across 434 wheat accessions. We scrutinised the most frequently occurring introgressions and identified potential donors. We also identified a large number of introgressions that are private to single genotypes. The practical value of the presented data sets was exemplified by QTL mapping of a recessive powdery mildew resistance gene on chromosome 7D embedded in a 14 Mb introgression that is only present in the Turkish cultivar Pamukale. Finally, we identified several duplicated segments in distal chromosomal regions, which we propose were the results of inter-homoeologue recombination. Our findings suggest that recombination between homoeologous chromosomes was a frequent event during wheat evolution.

## Materials and methods

### Mapping of exome capture and other reads

Genomes were first indexed with bwa index (v.0.7.17, Li 2013) using the –c flag. The exome capture reads were then mapped on the CS genome (IWGSC RefSeq v2.1) using bwa mem (v.0.7.17). The resulting sam file was then converted to .bam format, and duplicate reads were removed with samtools (v.1.6, Danecek et al. 2021) using the functions sort fixmate and markup. Coverage ratio was then calculated using the deeptools (v.3.5.1, Ramírez et al. 2016) command bamCompare, using the mapping of CS exome capture reads as normalisation control with the flags: –minMappingQuality 30, –operation ratio, –binSize 500000. For GBS and WGS of wild relatives, the same method was used but with a bin size of 2 Mb.

### Detection of introgressions

Introgressions were automatically detected by a custom Perl script (https://github.com/matthias-heuberger/heubiSOFT/blob/main/assign_introgressions_WW), which evaluated for each bin if the coverage was below 0.5. Adjacent bins below this threshold were connected to form one putative introgression region. Note that this means if two regions of low coverage are separated by a region with coverage ratio above 0.5, these introgressions are counted separately. They might, however, not be truly independent introgression events. To exclude potential false-positive signals from deletions, we removed introgressions with an average coverage of less than 0.03 across the entire length. This value was chosen because it is higher than the median value of the observed deletions due to inter-homoeologue recombination, but lower than the value for the known 1RS.1BL introgression originating from rye.

### Determination of uniqueness of introgressions

To distinguish whether introgressions are unique or shared between accessions, they were classified based on whether their start and end points were the same in multiple accessions. Unique introgressions of size 10 Mb or more were further scrutinised manually. To be counted as unique, either the start or the endpoint had to be at least 2 Mb from the start or endpoint of another introgression, or the coverage ratio across the entire introgression had to deviate at least ± 30% from other introgressions in the same region.

### Determination of gene coverage

To determine the coverage of genes in the exome capture sequencing data mapping coverage of Chinese Spring against itself was analysed using the program featureCounts (v.2.0.0, Liao et al. 2014). The average coverage across the entire gene was then calculated using the read counts per gene obtained by featureCounts and the gene and read length. A gene was counted as sufficiently covered, if the gene had an average coverage of ≥ 3 reads.

### Fungal material and infection tests

*Blumeria graminis* f. sp. *tritici* (*Bgt*) isolates Bgt_07004 and Bgt_10001 were used to evaluate Pamukale resistance. Both *Bgt* isolates were avirulent on Pamukale (no fungal growth) but virulent on the susceptible cultivars Frisal and Kanzler (leaves fully covered in fungal mycelia, in Fig. 3b; at least 3 biological replicates were tested per *Bgt* isolate). To assess the resistance response, 3-cm leaf fragments were placed on water agar containing 500 ppm Benzimidazole and spray-inoculated with *Bgt*. The infection rate was evaluated 7–9 days after infection and described as the percentage of leaf area covered by fungal mycelia (ranging from 0 being completely resistant to 100 being completely susceptible). Other *Bgt* isolates used for phenotyping have been selected from a worldwide collection described in Sotiropoulos et al. (2022).

### SNP based genotyping and filtering

Genomic DNA was extracted from leaf tissues of Frisal, Pamukale and the 133 F2 individuals resulting from their cross, using the CTAB method (Stein et al. 2001). Genotyping has been performed by SGS INSTITUT FRESENIUS GmbH TraitGenetics (https://sgs-institut-fresenius.de) with the Wheat Illumina Infinium 25K SNP Array. The SNP sequences were blasted against the genomes of 10 wheat cultivars (Walkowiak et al. 2020), and only the SNPs consistently mapping to the same chromosome for all tested wheat genomes were kept for further analyses. From the initial 24,146 SNP markers, 12,936 SNP markers passed this filtering step.

### Linkage map construction and QTL mapping

Further filtering and linkage map construction was done with the software QTL IciMapping, (v.4.2; https://isbreedingen.caas.cn/; Meng et al. 2015). First, SNP markers which were non-polymorphic or missing in the parents were removed by deletion using the “SNP” functionality. Redundant markers and markers with more than 50% missing rate were removed with the “BIN” functionality using the following parameters: *p* value: 0.01, missing values considered. In the “MAP” functionality, chromosome and physical positions have been considered for linkage map construction. The final linkage map consists of 2,058 SNP markers and is available in Supp. Table S5. QTL mapping was performed with the R package R/qtl (v1.6), as described by (Müller et al. 2022). Briefly, the function read.cross() was used to import map position, genotypic (Supp. Table S5) and phenotypic data (Supp. Table S4) of the Pamukale x Frisal cross. Subsequently, jittermap() and calc.genoprob() commands were used to filter the genotypic data before performing QTL mapping with scanone(, model = ”np”). The significance threshold was estimated with 500 permutations.

### Statistical analyses

Chi-square goodness-of-fit test has been performed with base R. Plots and other figures have been produced with R/RStudio (v.4.3.0 and 2023.03.1, respectively), using the package ggplot2 or base R.

### PCR amplifications across candidate breakpoints

The region ± 3 kb of the suspected breakpoint of both homoeologous regions was extracted from IWGSC RefSeq v2.1. The two homoeologous regions were then aligned using the Smith–Waterman algorithm (Smith and Waterman 1981). Regions of the alignment with homoeologue-specific SNPs or InDels were chosen as targets for primer design, which was carried out using Primer-BLAST (Ye et al. 2012) based on Primer3 (Untergasser et al. 2012). PCR amplifications were done using 50 ng of template DNA, using a homemade Pfux7 polymerase (Nørholm 2010) with the Phusion Green HC Buffer (F538L, Thermo Fisher). Primer sequences are listed in Supp. Table S7.

### Amplicon-sequencing

To demonstrate the presence of individual homoeologous genes from the three subgenomes, primers were designed, so that the primer sequences lie in a region that is conserved across the homoeologous genes, but the product contains homoeologue-specific SNPs. Primer sequences are listed in Supp. Table S7. PCR amplifications were performed as described above. Sequencing was performed as described in (Manser et al. 2024). Amplicons were purified with the E.Z.N.A. Cycle-Pure Kit (D6492-02, Omega Bio-Tek). The purified product (200 ng) was sent to Microsynth (Balgach, Switzerland). Adaptor-trimmed single reads were mapped to the reference coding sequences of Chinese Spring using blastn queries, and the best blast hit for each read was assigned to one of the triad genes.

### Identification of specific, shared transposable element insertions

Transposable element (TE) analysis was performed as described in Wicker et al. (2022). Briefly, individual *RLC_Angela* copies were extracted from the genomes of *T. timopheevii* (Grewal et al. 2024), Chinese Spring, Renan, Attraktion, Julius, Jagger and Lancer. The extracted copies were then aligned to the consensus sequence of *RLC_Angela*, and a variant call file (vcf) was constructed. Principal component analysis (PCA) was then done first on the entire dataset and then on sub-families identified in the first round of PCA. This was done to identify *RLC_Angela* copies that are specific to the tested genotypes. The genotype-specific copies were then used as markers for the candidate introgressions.

### Mitotic chromosome spreads preparation

Root tip meristem cells were synchronised using hydroxyurea, accumulated in metaphase using amiprophos-methyl (APM) and mildly fixed with formaldehyde as described by Cápal et al. (2023) and Doležel et al. (2023). The synchronised root tips were used to prepare chromosome spreads for genomic in situ hybridisation (GISH) using the drop technique demonstrated by Kato et al. (2004, 2006), with modifications as described by Said et al. (2018, 2019a, b, 2021). Briefly, synchronised roots of seedlings were treated with ice water overnight. Treated roots were excised (2 cm), fixed in ice-cold 90% acetic acid for 10 min, washed three times with ethanol 70% on ice and stored at − 20 °C until use. The roots were washed thrice with distilled water for 10 min and then rinsed in 1 × KCl buffer pH 4.0 for 5 min. The meristematic tissue of the root tips was dissected and digested in a 0.5 Eppendorf containing a 20 μl mix of 4% cellulase Onozuka R-10 (Yakult Pharmaceutical, Tokyo, Japan) and 1% Pectolyase Y-23 (Duchefa Biochemie, Haarlem, The Netherlands) in 1 × KCl buffer pH 4.0 solution for 58 min at 37 °C. Then, the reaction was stopped by filling the tube with TE buffer pH 7.6 for 5 min and then washing three times with 100% ethanol on ice. The root tips that had been digested were gently crushed using a rounded-off dissecting needle in a mixture of pure ice-cold acetic acid and methanol in a ratio of 9:1 v/v, respectively. The cell suspension was dropped onto glass slides (6–7 μl per slide) in a moist box and dried slowly under halfcover.

### Preparation of probes for GISH

To detect the A and D genomes, genomic DNA of diploid *Triticum urartu* acc. ECN01c106069 (2*n* = 2 × 14, AA) obtained from Crop Research Institute (Prague, Czechia) and diploid *Aegilops tauschii* acc. MVGB605 (2*n* = 2 × 14, DD) provided by Martonvásár Cereal Genebank (Martonvásár, Hungary) were labelled with digoxigenin and biotin, respectively, by nick translation using standard kits of Nick Translation Mix (Roche, Mannheim, Germany) following the manufacturer’s instructions.

### Fluorescence in situ hybridisation

Labelled probes for GISH were localised following the protocols of Said et al. (2018, 2019a, b, 2021) with modifications. Briefly, digoxigenin-labelled probes were detected using anti-digoxigenin fluorescein isothiocyanate (Roche). Biotin-labelled probes were detected with Cy3-conjugated streptavidin (Invitrogen, Life Technologies, Carlsbad, USA). The hybridisation mixture (total volume = 10 μl/slide) contained 50 ng labelled probe DNA, 50% v/v formamide, 2 × SSC (0.15 mol/l NaCl plus 0.015 mol/l sodium citrate), 10% w/v dextran sulphate, 0.4 μg salmon sperm DNA and 0.1% w/v sodium dodecyl sulphate. An amount of 5 μg genomic DNA of *Ae. speltoides* MvGB1321 (2n = 2 × 14, BB) obtained from Martonvásár Cereal Genebank was included in the hybridisation mix as blocking DNA. The chromosomes and probes were denatured simultaneously at 80 °C for 3 min under high moisture conditions. The hybridisation was carried out overnight at 37 °C under humid conditions. The slides were washed, the hybridisation sites were detected, and chromosomes were mounted and counterstained with 4′,6-diamidino-2-phenylindole (DAPI) in Vectashield media (Vector Laboratories, Burlingame, USA).

### Microscopy, software, signal capture and image analysis

Chromosome preparations were examined using an Axio Imager Z.2 Zeiss microscope (Zeiss, Oberkochen, Germany) equipped with a Cool Cube 1 (MetaSystems, Altlussheim, Germany) camera and appropriate optical filter sets. The signal capture and image processing were performed using ISIS software (MetaSystems).

## Results

### A catalogue of wheat chromosomal introgressions

We used exome capture data from the 434 wheat accessions from the WHEALBI collection as a basis to identify introgressions. Exome capture data used here were generated using oligomeric capture probes designed from the gene annotation of the first complete assembly of the wheat (cv. Chinese Spring) genome assembly (Jordan et al. 2015; Warr et al. 2015; Pont et al. 2019). The exome capture data cover ~ 1% of the genome and ~ 59.8% of all annotated genes (Supp. Table S1). To identify candidate introgression regions, exome capture sequence reads from each wheat accession were mapped against IWGSC RefSeq v2.1 (cv. Chinese Spring, hereafter referred to simply as “Chinese Spring” or CS). The resulting read coverage was normalised against the sequence coverage of reads from CS (i.e. the ratio between the coverage of the respective accession and the Chinese Spring reference, hereafter referred to as “coverage ratio”). In regions where a sample and the reference have the same haplotype, a coverage ratio of ~ 1 was expected, while a read coverage of 0 would indicate a highly divergent haplotype where no reads map. In case of duplicated segments, read coverage is approximately 2 (see below). Because a very small fraction of exome capture probes represent repetitive DNA which can lead to a very high coverage ratio, we set the upper cut off to 2 for the heatmap plots. We defined introgressions as regions showing a coverage ratio smaller than 0.5 (i.e. the coverage was only one-half or less than CS in the same bin) and higher than 0.03 to reduce false positives from deletions. Adjacent chromosome bins which were classified as introgressions were merged for determining the total number of introgressed segments. With this method, introgressions were defined by comparison with CS and it was not possible to determine whether the introgression was present in the specific accession or in CS. One example of an introgression in CS has been described on chromosome 6A and it originates from *T. monococcum* (Ahmed et al. 2023).

**Fig. 1:**
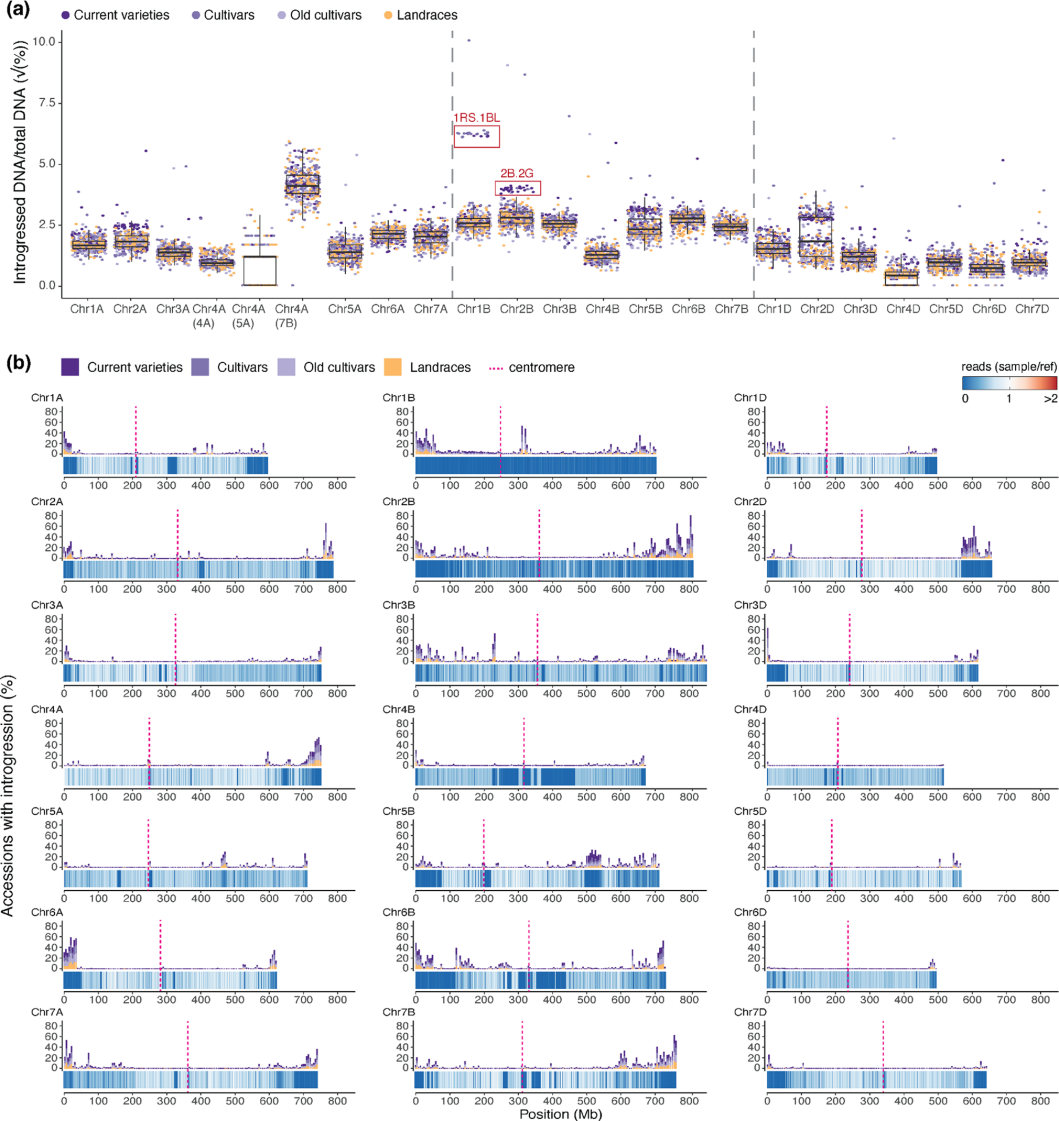
Overview of chromosomal introgressions in the WHEALBI collection. **a** Boxplot showing the percentage of each chromosome that is part of an introgression in hexaploid genotypes of the WHEALBI collection. Each dot corresponds to one wheat accession. Colour indicates the group. Chromosome 4A was split into three parts, according to the subgenome ancestry of the different segments of chromosome 4A (Dvorak et al. 2018). Boxes indicate the inter-quartile range (IQR) with the central line indicating the median and the whiskers indicating the minimum and maximum values without outliers extending beyond—1.5*IQR and maximum + 1.5*IQR. **b** Introgression frequencies along wheat chromosomes in 500 kb bins. For each chromosome, the barchart on top shows the percentage of accessions having an introgression in each bin. The heatmap at the bottom shows the coverage of the accession with the lowest coverage for each respective bin. For example, in the case of Chr. 1B, there is one accession in which the entire chromosome is replaced by Chr. 1R from rye. The top track, however, shows that introgressed 1R segments are generally rare in the wheat accessions tested

Using the coverage ratio cut-off of < 0.5, we identified 24,981 putative introgressions of at least 2 Mb in size in the WHEALBI collection (i.e. four adjacent genomic bins, see methods). Introgressed regions span, on average, 3.96% of the genome of a given accession (median = 3.81%). The D subgenome has the smallest introgressed fraction with an average of 1.73% and a median of 1.67% (Fig. 1a). The median number of introgressions per accession is 55 (mean = 57.69), eight are larger than 5 Mb (mean = 9.00), and three are larger than 10 Mb (mean = 2.84). Strikingly, 393 of 433 accessions have five or more introgressions larger than 5 Mb and more than half (50.35%) of all accessions have at least three introgressions larger than 10 Mb. Finally, 2933 introgressions are private to single accessions, of which 864 are at least 5 Mb in size and 118 are at least 10 Mb (of which 10 have a coverage ratio < 0.03, Supp. Table S2). Overall, these data indicate that multiple and large introgressions are common in accessions of the WHEALBI collection.

### Large chromosomal introgressions are frequent in the WHEALBI collection

**Fig. 2:**
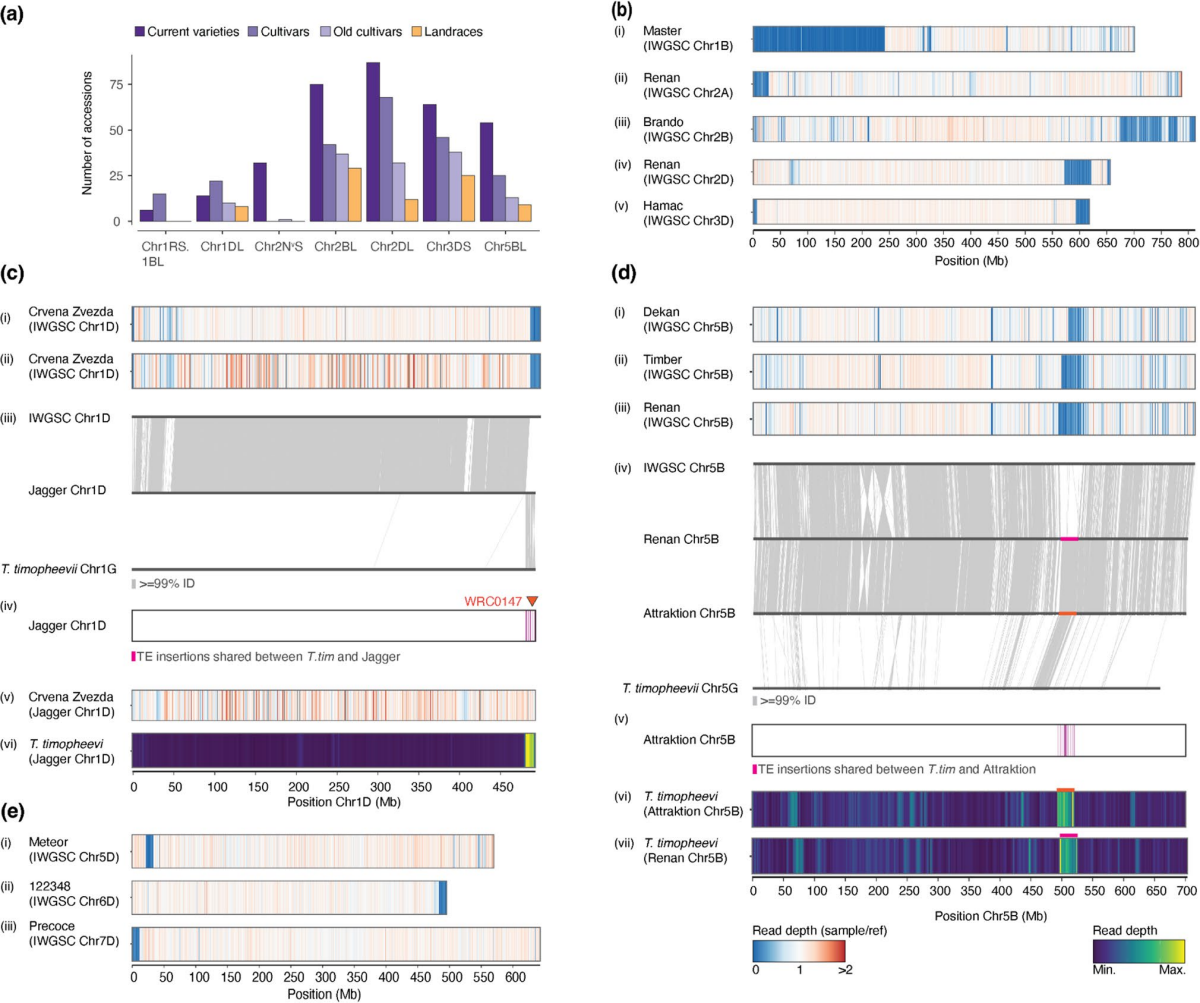
Detailed characterisation of chromosomal introgressions **a** Barplot showing the number of accessions carrying introgressions. This includes the Chr. 1RS.1BL translocation, the *Ae. ventricosa* introgression on Chr. 2A, the *T. timopheevii* introgression on Chr. 2B, the *Ae. markgrafii* introgression on Chr. 2D, the *Ae. comosa*/*Ae. uniarista* introgression on Chr. 3D (Keilwagen et al. 2022) and the two herein described introgressions likely coming from *T. timopheevii* on Chr. 1D and Chr. 5B. **b** Introgression regions quantified in  panel **a**. Coverage of normalised exome capture reads of: (i) Master on Chr. 1B; (ii) Renan on Chr. 2A; (iii) Brando on Chr. 2B; (iv) Renan on Chr. 2D; and (v). Hamac on Chr. 3D. **c** Candidate introgression region on Chr. 1D; (i) Coverage of normalised exome capture reads of Crvena Zvezda on Chr. 1D; (ii) coverage of normalised GBS reads of Crvena Zvezda on Chr. 1D; (iii) chromosomal synteny between CS Chr. 1D and Jagger Chr. 1D; and (iv) *RLC_Angela* TE copies specific to *T. timopheevii* and Jagger. The position of the KASP marker WRC1047 is indicated by a triangle (v) coverage of normalised GBS reads of Crvena Zvezda on Jagger Chr. 1D; (vi) *T. timopheevii* WGS reads mapped on Jagger Chr. 1D. **d** Candidate introgression region on chromosome 5B. Normalised coverage of: (i) Dekan exome capture reads; (ii) Timber exome capture reads and (iii) Renan exome capture reads mapped on CS Chr. 5B. (iv) Chromosomal collinearity between Chinese Spring, Renan, Attraktion and *T.* *timopheevii* chromosome 5B. (v) *RLC_Angela* TE copies specific to *T. timopheevii* and Attraktion. *T. timopheevii* WGS reads mapped on chromosome 5B in Attraktion (vi) and Renan 5B (vii). **e** Examples of rare introgressions. Coverage of normalised exome capture reads of: (i) Meteor on chromosome 5D; (ii) 122,348 on chromosome 6D; (iii) Precoce on chromosome 7D. Values for exome capture were calculated in 500 kb bins, GBS and WGS in 2 Mb bins

We identified several introgressions from known donor species previously described in the literature (Fig. 2a–b). The largest introgression was in Riebesel St. 47-51 (WW-098), where the entire 700 Mb chromosome 1B is replaced by *S. cereale* (rye) chromosome 1R (727 Mb) (Friebe et al. 1987). Among the introgressions larger than 10 Mb, the most frequently observed is located on chromosome 2D from ~ 573 Mb to ~ 622 Mb (Figs. 1b, 2a, Supp. Fig. 1a). This introgression has been identified in multiple reference-quality genome assemblies (RQAs) and has been suggested to originate from *Aegilops markgrafii* (Walkowiak et al. 2020; Keilwagen et al. 2022; Supp. Fig. 1a). Strikingly, this introgression is found in the majority of current varieties and cultivars (87.9% and 52.3%, respectively), but only in 12.6% of landraces. This introgression was previously reported to be enriched in elite genotypes and positively affects total plant biomass and number of grains per m^2^ under optimal conditions (Voss-Fels et al. 2019; Keilwagen et al. 2022). It was also shown to contain a homologue of a gene conferring resistance to yellow rust (Keilwagen et al. 2022).

Another large introgression with a known donor species is found only in current varieties (32.3%), except for one old cultivar (Taferstat, WW-420). It is located in a ~ 29 Mb segment at the end of chromosome arm 2AS (Fig. 2a). This introgression was previously described as “2NS” and originates from *Ae. ventricosa* (Walkowiak et al. 2020). It contains a source of wheat blast resistance, as well as a gene cluster containing the rust resistance genes *Lr37*, *Yr17* and *Sr38* (Bariana and McIntosh 1993; Helguera et al. 2003; Cruz et al. 2016). To verify that the introgression detected in our analysis corresponds to the 2NS translocation, we mapped whole-genome sequencing (WGS; Walkowiak et al. 2020) reads onto the genome of cultivar Renan (Aury et al. 2022), which, based on our analysis, contains the 2NS introgression. Indeed, the *Ae. ventricosa* reads show a coverage maximum at the position of the suspected introgression, confirming previous reports of cv. Renan carrying this introgression (Supp. Fig. 1b, Aury et al. 2022). We emphasise that the origin of this introgression from *Ae. ventricosa* has been confirmed in previous studies using multiple different methods (Walkowiak et al. 2020).

An introgression with unclear origin common to 54 accessions is present in chromosome arm 1DL with a size of ~ 11.6 Mb. Comparison of 1D chromosomes with reference-quality assemblies (RQAs), showed that wheat cv. Jagger has an abrupt loss of sequence collinearity with CS at around position ~ 487 Mb (Fig. 2c). To investigate the origin of the introgression found in the WHEALBI accessions, we used genotyping-by-sequencing (GBS) data of three accessions predicted to contain the introgression (cv. Crvena Zvezda, Spada and Blueboy, Schulthess et al. 2022) and mapped them to both Jagger and CS. The GBS data confirmed that Crvena Zvezda, Spada and Blueboy have an introgression at the distal end of chromosome 1DL starting at ~ 487 Mb (Fig. 2c, Supp. Fig. 2) when using CS as reference. However, when mapping the GBS data to Jagger, we found no reduced sequence coverage in this region, indicating that all four cultivars share the same introgression. Additionally, Illumina reads from *T. timopheevii* (Walkowiak et al. 2020) mapped at a high coverage in the region of the candidate introgression in Jagger (Fig. 2c), which is in line with a recent publication (Coombes et al. 2024). We also identified transposable element insertions specific to *T. timopheevii* and Jagger in the region of the introgression (Fig. 2c). Furthermore, blastn queries using the *T. timopheevii*-specific KASP marker WRC0147 (King et al. 2022) showed that Jagger has the *T. timopheevii* variant within the introgression. Lastly, alignment of the Chr. 1D of Jagger and Chr. 1G of *T. timopheevii* (Grewal et al. 2024) shows high similarity between the region of the introgression in Jagger and the terminal region of the long arm of Chr. 1G. From this, we conclude that the donor of the 1D introgression is *T. timopheevii* or a species closely related to it.

A previously described introgression with an unknown donor (Cheng et al. 2019; Kale et al. 2022; Schulthess et al. 2022) is present in 101 accessions (54 of which are current varieties). This introgression of ~ 30 Mb is located in chromosome 5B. Interestingly, the size of the introgressed fragment varies between accessions. The longest version was found in cv. Renan, which is 30 Mb (Fig. 2d, replacing a 37.5 Mb segment in CS). This introgression is also present in the chromosome-scale assemblies of Fielder, Mace, Jagger, Stanley and Attraktion. Notably, they have two different versions of the introgression, both shorter than the version found in Renan (Fig. 2d, Supp. Fig. 3).

To identify potential donor species for the introgression, we mapped Illumina reads from *T. timopheevii*, *Ae. ventricosa*, *T. ponticum* and *Ae. speltoides* against the genomes of Renan and Attraktion. WGS reads originating from *T. timopheevii* showed a high sequence coverage in the putative introgression regions for Renan and Attraktion (Fig. 2d) which was not the case for the other wheat relatives (Supp. Fig. 4, Supp. Fig. 5). Coincidentally, Attraktion has another introgression which is reported to originate from *T. timopheevii* on chromosome 2B (Walkowiak et al. 2020; Kale et al. 2022). We made use of this and compared the coverage of *T. timopheevii* reads between the introgression of chromosome 2B and chromosome 5B found in Attraktion (Supp. Fig. 6). The median *T. timopheevii* read density in the introgressions on chromosome 2B and chromosome 5B is highly similar (61,723 vs. 61,705 reads/Mb), and much higher than in the rest of the genome (median = 4,246 reads/Mb). This strongly suggests that the introgression on chromosome 5B comes from a closely related (if not the same) donor species as the one on chromosome 2B, which is accepted to stem from *T. timopheevii*. Again, we identified transposable element insertions specific to *T. timopheevii* and Attraktion in the region of the introgression (Fig. 2d). Blastn queries using the *T. timopheevii*-specific variant of the KASP marker WRC0646 (King et al. 2022) showed perfect alignment with Renan (but not Attraktion, because Renan and Attraktion differ in the size of the introgression and the position of the marker lies outside the introgressed region in Attraktion). Furthermore, alignment of the Chr. 5B of Attraktion and Chr. 5G of *T. timopheevii* (Grewal et al. 2024) shows high similarity between the region of the introgression in Attraktion and the syntenic region of the long arm of Chr. 5G.

The 5B introgression in Renan contains 307 genes including two NB-ARC LRR (NLR) resistance gene analogues. One of them lies in a cluster of 24 Auxin responsive SAUR genes, which were previously implied to show a role in drought tolerance (He et al. 2021). A full list of genes found in this introgression can be found in Supp. Table S3.

While the more frequent introgressions described above are often documented sources for one or multiple known beneficial traits in the wheat gene pool, this is not known for rare introgressions. We found such introgressions, for example, in the accession Meteor on chromosome 5D spanning ~ 21.5–33 Mb in CS, on 6D in the accession 122,348 (~ 483–495.3 Mb) and on 7D in the accession Precoce (telomere—11.5 Mb, Fig. 2e). In Chinese Spring, the segments replaced by these rare introgressions contain 9 (Meteor_5D), 50 (122348_6D) and 81 (Precoce_7D) NLR genes. As introgressions often replace homoeologous segments, it is conceivable that these introgressions introduce new NLR diversity, originating from wild relatives into the wheat gene pool.

### An introgression in cv. Pamukale contains a QTL for powdery mildew resistance

**Fig. 3:**
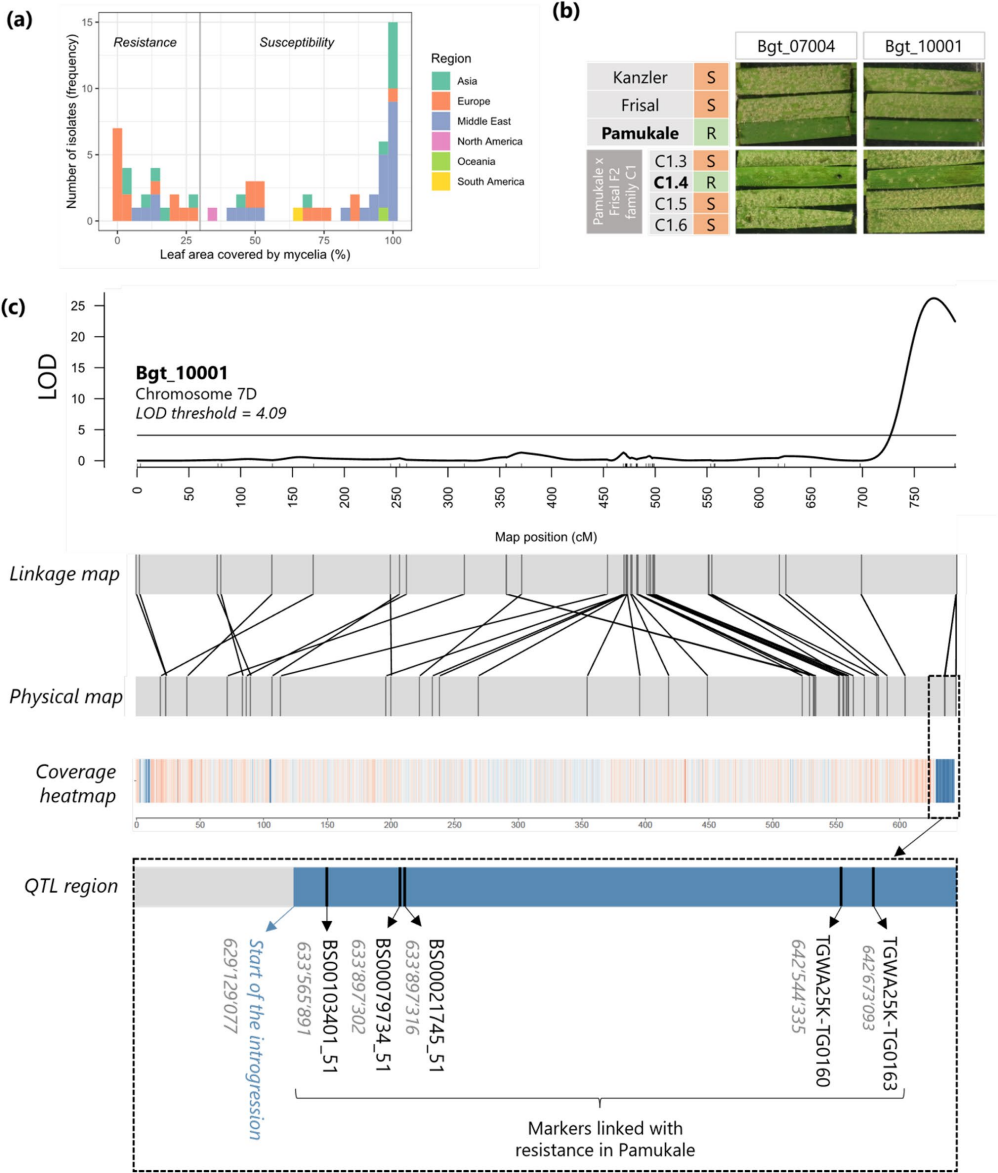
A QTL for powdery mildew resistance in cv. Pamukale is located within an introgression in chromosome 7D. **a** Frequency of resistant and susceptible phenotype against 66 *Bgt* isolates from a worldwide collection. A leaf coverage below 30% corresponds to a resistant phenotype, while above 30% is considered susceptible. **b** Pamukale provides seedling stage resistance against the *Bgt* isolates Bgt_07004 and Bgt_10001. The F2 progeny individuals (lower panel) show a recessive segregation of the resistance phenotype. The phenotype was evaluated eight days after spray inoculation of detached leaf segments. **c **A QTL locus for powdery mildew resistance in chromosome 7D is linked with five SNP markers located in an alien introgression unique to the cultivar Pamukale. The representation of the *R* gene locus is not in scale

Another example of a rare, previously undescribed introgression is found on chromosome 7D, private to cv. Pamukale (WW-444). Since previously described introgressions were often linked with pathogen resistance, for example, the 1RS.1BL translocation (Crespo-Herrera et al. 2017), we considered Pamukale as an interesting cultivar for mapping novel powdery mildew resistance (*R*) genes. Phenotyping on Pamukale with 66 wheat powdery mildew (*Bgt)* isolates from a worldwide collection (Sotiropoulos et al. 2022) identified resistance to 24 *Bgt* isolates from different regions of the world (Fig. 3a). To map the underlying *R* locus, we crossed Pamukale with cv. Frisal, which is broadly susceptible to *Bgt* at the seedling stage (Fig. 3b). An F2 population inoculated with isolate Bgt_10001 segregated 40 resistant: 93 susceptible (chi^2^_1:3_ = 1.827; *P*_1df_ = 0.1765) indicating that resistance was conferred by a single recessive locus. In a separate test with *Bgt* isolate Bgt_07004, similar results were obtained, with 34 F2 individuals which were scored resistant and 99 susceptible (Supp. Table S4). The difference in ratio was attributed to error or quantitative variation in phenotyping.

All Pamukale x Frisal F2 individuals were genotyped with a 25 K SNP wheat array and a linkage map with 2,058 polymorphic markers distributed across the whole genome was constructed (Supp. Table S5). The linkage map was used for QTL mapping, which revealed a QTL for powdery mildew resistance to both *Bgt* isolates, located at the end of the long arm of chromosome 7D (Fig. 3c, Supp. Fig. 7), colocalising with the 7D introgression unique to Pamukale. The underlying gene in the QTL was named *PmPam*.

The QTL was flanked by the markers GENE-4442_121 and BS00103401_51, with the latter being completely linked with *PmPam* (LOD > 20; Fig. 3c). There were four additional SNP markers located in the same position as BS00103401_51 in the linkage map, which were perfectly linked (except for a few missing SNP marker calls). Interestingly, we observed that they were physically apart from each other, ranging from 633,565,891 to 642,673,093 in the CS reference genome (Fig. 3c). This clustering in the linkage map was attributed to the presence of the introgression in Pamukale (starting at position ~ 629.1 Mb) suppressing recombination in that genomic region. We therefore assume the QTL spanned the entire 14 Mb of the introgression, making fine mapping of *PmPam* nearly impossible (Fig. 3c).

*Pm5* is a recessive *R* allelic series located at the end of chromosome 7B (Xie et al. 2020) and has homoeologues in chromosomes 7A and 7D. To exclude that *PmPam* was derived from the *Pm5* locus, we performed a synteny comparison between the ends of chromosomes 7A, 7B and 7D and found that in chromosome 7D the *Pm5* homoeologue is located at 616,968,098 in the CS genome (Supp. Fig. 8). It is therefore not in the introgression region, which starts at 629,129,077, and we concluded that *PmPam* is distinct from *Pm5* and its homoeologues.

### Multiple wheat accessions show evidence for inter-homoeologue recombination

**Fig. 4:**
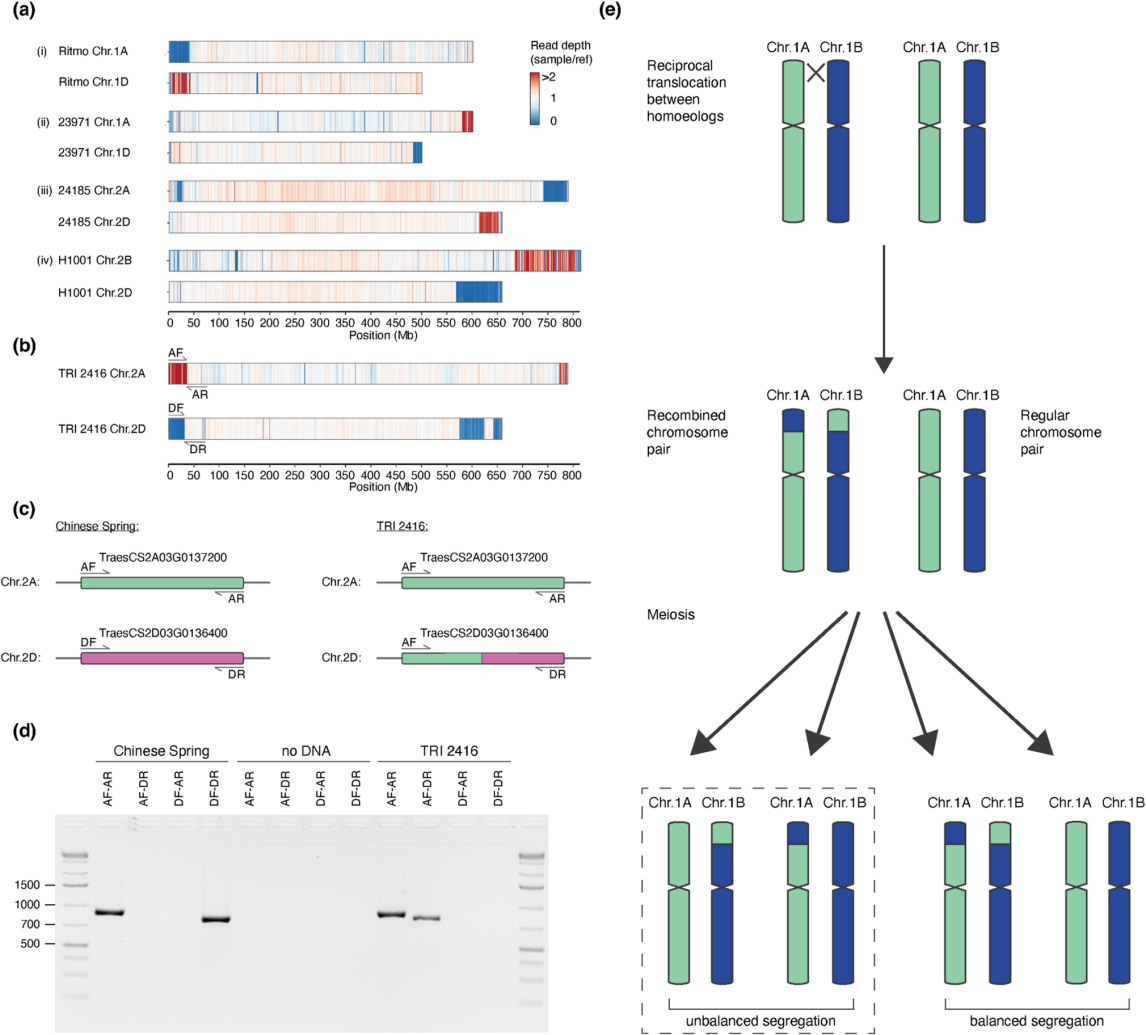
Evidence for recombination between homoeologous chromosomes in some genotypes of bread wheat. **a** and **b** show examples of putative inter-homoeologue recombination events. The heatmap shows coverage of normalised exome capture reads mapped against CS. Values are calculated in 500 kb bins. **b** An example used for PCR verification. Location of primers is indicated and shown in more detail in panel **c**. **c** Scheme of primers designed to confirm inter-homoeologue translocation/duplication by PCR across the translocation breakpoint. The breakpoint lies within the homoeologous genes *TraesCS2A03G0137200* and *TraesCS2D03G0136400*. **d** 1.2% Agarose gel loaded PCR products. There is a PCR product of the expected size for the primer combinations AF-AR and DF-DR in CS. The DF-DR product is absent in TRI 2416, and there is amplification for AF-DR. **e** Schematic model of reciprocal translocations between homoeologues. Translocation is shown with the example of chromosomes 1A and 1B. After recombination, meiosis leads to the segregation of homoeologous chromosomes, in which two gametes contain unbalanced chromosomal content

We found chromosomal segments in multiple accessions with a relatively even coverage ratio of ~ 2, but there was a concomitant absence of reads in the corresponding segment on one of the homoeologous chromosomes. One example is the modern variety Ritmo (WW-150, Fig. 4a) where the first ~ 39 Mb of chromosome 1A have almost no read coverage (median = 0.03), whereas the first ~ 41 Mb of chromosome 1D have a median coverage ratio of 1.87. This indicates that the first ~ 39 Mb of chromosome 1A in Ritmo are missing, while the beginning of chromosome 1D is duplicated. An explanation of such an observation is a reciprocal translocation between homoeologous chromosomes. Reciprocal translocations were also described in rice (Wicker et al. 2015), synthetic allopolyploid wheat (Zhang et al. 2020) and more recently in newly produced *Am. muticum*/hexaploid wheat introgression lines (Coombes et al. 2023). In the case of Ritmo, we propose that chromosome 1D is “regular”, whereas the distal end of chromosome arm 1AS was replaced by the first 41 Mb of chromosome 1D, which would therefore occur twice in the genome of Ritmo (Fig. 4).

Another example was found in the Albanian traditional cultivar TRI 2416 (WW-078, Fig. 4b), where the first 36 Mb of chromosome 2A have a median coverage ratio of 2.208, whereas the first 31 Mb of chromosome 2D have almost no mapped reads (median = 0.017). We propose that chromosome 2A is “regular”, while the distal end of chromosome arm 2DL was replaced by the first 36 Mb of chromosome 2A, which therefore occurs twice in the genome of TRI 2416. To confirm this rearrangement, we first identified the putative translocation breakpoints bioinformatically by identifying regions where Illumina reads map on chromosome 2A, and their mate reads map on chromosome 2D. We identified the candidate breakpoints to be in the homoeologous genes *TraesCS2A03G0137200* (chromosome 2A) and *TraesCS2D03G0136400* (chromosome 2D, respectively) (Supp. Fig. 9). Indeed, we found sequencing reads covering the recombination breakpoint (Supp. Fig. 9). We then designed primers, based on the CS genome, and performed PCR amplifications across the breakpoint that confirmed the proposed arrangement (Fig. 4d). This indicates that the breakpoint of the 2A-2D translocation found in TRI 2416 indeed lies in the genes *TraesCS2A03G0137200* and *TraesCS2D03G0136400*. Based on 2A- and 2D-specific SNPs, we postulate the breakpoint to lie between the genomic positions 35,685,613 and 35,685,702 bp of chromosome 2A (31,143,922–31,144,011 bp in chromosome 2D, Supp. Fig. 9b). We further designed primers to amplify three homoeologous gene triads located in the proposed region of homoeologous exchange. The PCR product was then sequenced using Amplicon-sequencing (Manser et al. 2024). This showed complete absence of reads representing the D genome gene copy, while reads from the A gene copy were over-represented (Supp. Fig. 10a).

**Fig. 5:**
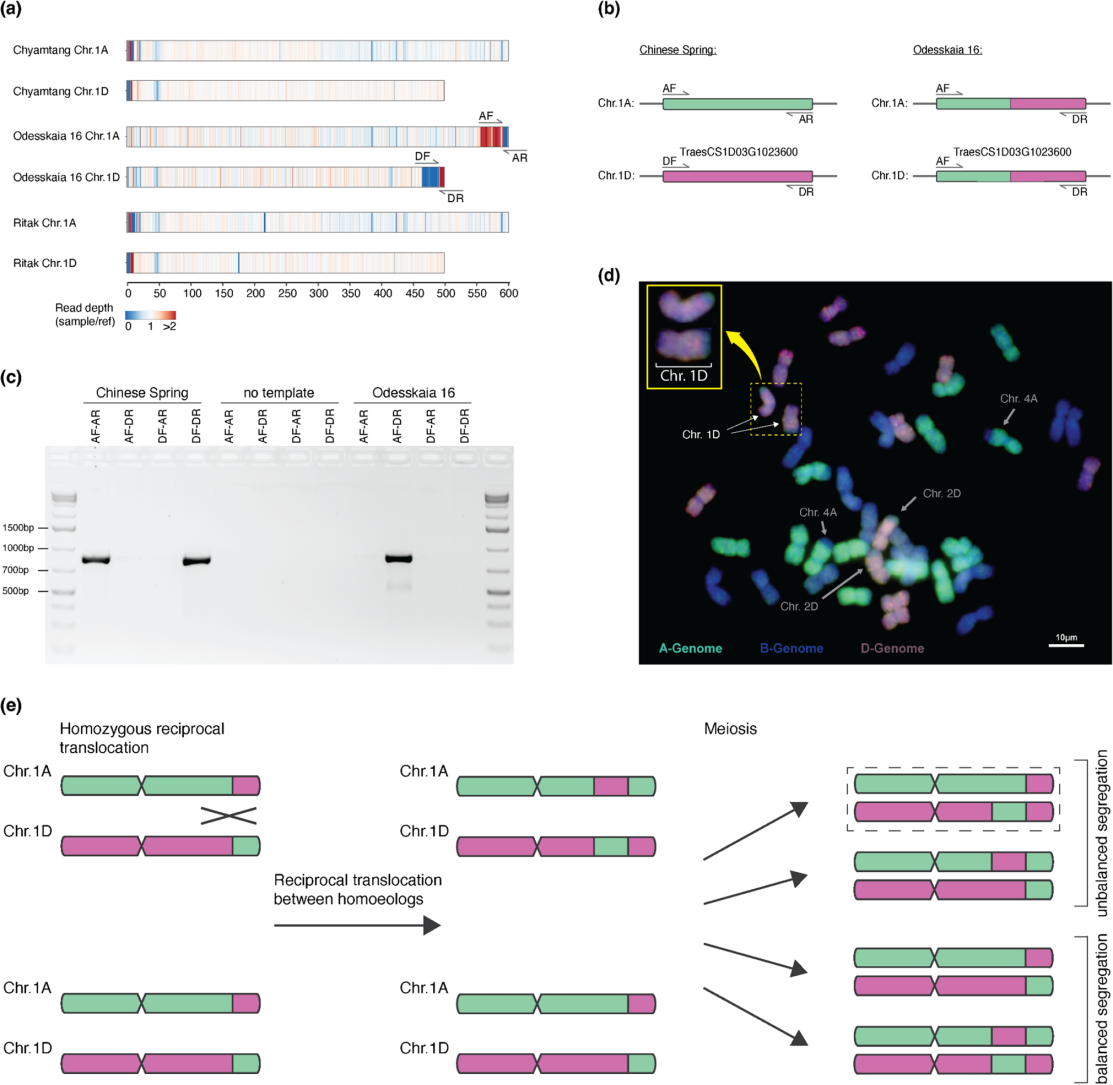
Evidence for recurrent recombination between homoeologous chromosomes in some genotypes of bread wheat. **a** Examples of suspected double inter-homoeologue recombination events. Heatmap showing coverage of normalised exome capture reads mapped on the CS genome. Values are calculated in 500 kb bins. Location of primers are indicated and shown in more detail in: **b** Scheme of primers designed for PCR amplification across the suspected translocation breakpoint, which was within gene *TraesCS1D03G1023600*. **c** 1.2% agarose gel loaded with PCR products. **d** GISH on mitotic metaphase chromosome spreads prepared from root tips of wheat line Odesskaia 16. Genomic DNA probes of *T. urartu* (green) *Ae. tauschii* (red) and *Ae. speltoides* (blue) were applied to detect A, B and D genomes, respectively. Chr. 1D (white arrows) showing a terminal segment of A genome origin (green) is also enlarged in the top left. The known 4A/7B translocation is seen by a terminal segment of B genome origin (blue), and the introgression of *Ae. markgrafii* origin on Chr. 2D is seen by blue/green colour on the terminal segment of the long arm. **e** Schematic model of reciprocal translocations between homoeologues, starting with a heterozygous, single inter-homoeologue translocation carrier. Translocation is illustrated between chromosomes of the A and D genomes. Further recombination generates recombined chromosomes. Chromosome structure, as seen in Odesskaia 16, is shown with a dotted rectangle

Based on these results, we propose a model for inter-homoeologous exchange similar to what has been proposed in rice (Wicker et al. 2015, Fig. 4e): the first step is a symmetric reciprocal translocation between homoeologous chromosomes (1A and 1B in the example in Fig. 4e). At meiosis, there are then four possible gametes, two with balanced chromosome content and two with a duplication of one homoeologue sequence and a loss of the other. Descendants of these cell lines can then, in later generations, become homozygous.

Interestingly, we also found cases where a subtelomeric region shows coverage ratio ~ 2, while the adjacent telomeric segment has a coverage ratio of ~ 0, with the pattern being inverted on one of the homoeologous chromosomes (Fig. 5a). This is especially prominent in the accession Odesskaia 16 (WW-403), where a 38 Mb (554–592 Mb) segment on the long arm of chromosome 1A shows a median coverage ratio of 2.08, and the adjacent (telomeric) 6.5 Mb have a median coverage ratio of 0.015. A homoeologous segment on chromosome 1D (464–491 Mb) shows a median coverage ratio of 0.027, whereas the telomeric 7.5 Mb has a median coverage ratio of 2.37 (Fig. 5a). Expanding the model of inter-homoeologous exchange, we propose that these examples resulted from two recombination events (Fig. 5e): the evolution of the Odesskaia 16 lineage started having a homozygous translocation in which the telomeric 7.5 Mb of chromosome 1D and the telomeric 38 Mb of chromosome 1A were swapped (Fig. 5). Subsequently, a second, larger inter-homoeologue recombination exchanged the telomeric ~ 50 Mb between the two recombinant chromosomes. As for the model in Fig. 4e, unbalanced segregation led to the duplication/deletion pattern observed in Odesskaia 16.

**Fig. 6:**
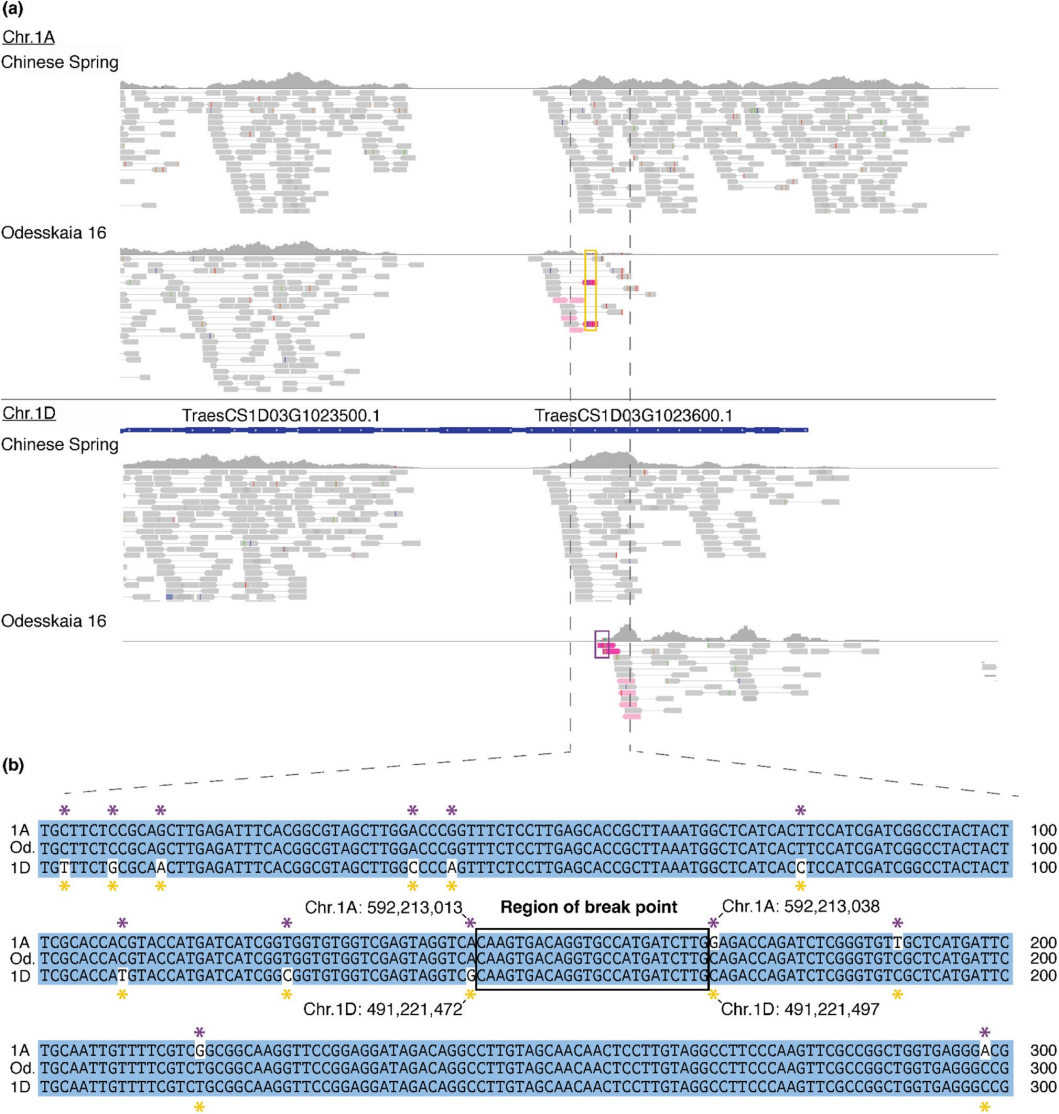
Identification of inter-homoeologue recombination breakpoint. **a** Visualisation of mapped reads from Chinese Spring and Odesskaia 16 on CS exported from IGV (Robinson et al. 2011). Mate-pairs going across the breakpoint (one mate mapping to Chr. 1A the other to Chr. 1D) were coloured in light pink. Reads that show both 1A- and 1D-specific SNPs in the same read (i.e. chimeric reads) are coloured in pink and framed by either yellow or purple rectangles. **b** Alignment of Chr. 1A and Chr. 1D in the region surrounding the suspected breakpoint 1A- or 1D-specific SNPs are indicated by asterisks. There were no reads on either 1A or 1D in this segment, where the corresponding mate mapped to the syntenic position in 1B

We again used information from paired-end Illumina reads to identify the precise translocation breakpoints. Although the proximal breakpoint could not be identified, either because the gene containing the breakpoint was not sequenced using exome capture (due to only 59.8% of genes being sequenced or because the breakpoint does not lie within a gene), we could estimate, based on sequence coverage data, the breakpoint to lie between the region corresponding to 463,719,382 and 463,727,141 in CS chromosome 1D. The distal breakpoint was found in the gene *TraesCS1D03G1023600*. Again, we found forward and reverse read pairs covering the recombination breakpoint (Fig. 6). This chromosomal position had double the coverage on chromosome 1A proximal to the breakpoint, and no coverage distal of the breakpoint on and, complementary, no coverage on chromosome 1D before the breakpoint and double the coverage after the breakpoint (Fig. 6a). We used homoeologue-specific PCR to confirm this breakpoint; the primer combinations AF-AR and DF-DR gave a PCR product of the expected length (832 bp), when using genomic DNA of CS (Fig. 5c). In the case of Odesskaia 16, PCR amplification occurred only for the primer combination AF-DR. We again designed primers for PCR amplification of three homoeologous triads, which were sequenced with Amplicon-sequencing. As for TRI 2416, there were no sequence reads representing the D genome copy, while reads for the A genome copy were over-represented (Supp. Fig. 10b). We further performed genomic in situ hybridisation (GISH) with subgenome-specific DNA probes. This showed a segment of A genome origin on the long arm of chromosome 1D (Fig. 5d), in line with the hypothesis that this segment is duplicated from chromosome 1A. The same experiment for TRI 2416 was, however, not conclusive, as the observed segment of A genome origin on chromosome 2D was substantially smaller than expected (data not shown).

From these data, we conclude that the WHEALBI collection contains multiple wheat accessions which contain duplicated chromosome segments that resulted from inter-homoeologue recombination followed by unbalanced segregation (Fig. 5e).

## Discussion

In this study, we exploited the public resource of exome capture data from a worldwide collection of 434 hexaploid wheat accessions, which includes currently used varieties, old cultivars and landraces. Although the exome capture data only represented about 59.8% of all genes and less than 1% of the genome, the data proved to be highly useful for the automated identification of introgressed chromosomal segments. Using the straightforward approach of analysing sequence read coverage normalised by reads of a reference, we shed light on the diverse introgression landscape found in the WHEALBI collection (Pont et al. 2019), composed of over 24,981 introgressions.

Wheat breeding depends on diversity in the gene pool. The very high number of candidate introgressions identified here indicates that a considerable part of the diversity in the wheat gene pool derives from such events. While some of the introgressions were made by humans, many may in fact be the result of substantial and natural gene flow.

These numbers are congruent with findings of a previous study that identified hundreds of introgressions based on 10 RQAs (Walkowiak et al. 2020), but in this study we went a step further, providing an introgression atlas of wheat material used in current breeding programmes. Nonetheless, the numbers presented in this study are likely an overestimation, because in our method regions of low coverage must be continuous. This means that, for example, if an internal segment of an introgression recombines and is replaced by common wheat, the internal segment will have a normal coverage ratio of ~ 1 and the introgression will therefore be split in two (Zhou et al. 2020). Our method will then count this as two introgressions, although it came from a single event.

The chosen approach allowed us to assess the frequencies of previously known major introgressions in the wheat gene pool. We could, for example, confirm the prevalence of an introgression from *Ae. markgrafii* found on chromosome 2D in western breeding material (Walkowiak et al. 2020; Keilwagen et al. 2022). Strikingly, more than half of all accessions had multiple introgressed segments of 10 Mb or larger, with smaller introgressions being even more prevalent. The data from introgressions that are shared between multiple accessions indicates that introgressed segments are often partially removed, likely through back-crosses with lines which do not contain them (Zhou et al. 2020). One example is the 5B introgression shown in Fig. 2d. Therefore, it is often not possible to determine whether an introgression happened only once and was later partially lost, or whether a similar fragment was introgressed multiple times. Despite the difficulties in estimating how frequently alien chromosome segments are introgressed, our data show that almost all wheat accessions studied here contain a complex mosaic of chromosomal segments coming from different genetic backgrounds.

### Diverse donor species contribute potentially important genetic material

We could identify the potential donor species for a few major introgressions. This is, in principle, only possible if genomic sequence data of the donor species are available. Indeed, we confirmed *Ae. ventricosa* as the donor species for the previously described introgression on chromosome 2A (Walkowiak et al. 2020; Keilwagen et al. 2022). More importantly, we identified *T. timopheevii* as the putative donor of a previously described 5B introgression (Cheng et al. 2019; Kale et al. 2022; Schulthess et al. 2022). We used publicly available Illumina reads from *T. timopheevii*, *Ae. ventricosa*, *Th. ponticum* and *Ae. speltoides*, which showed that *T. timopheevii* is the best candidate donor because its reads mapped much more frequently and with higher quality to wheat accessions which contain the introgression. However, since read mappings were not perfect, it is possible that the introgression came from a different haplotype or a close relative of *T. timopheevii*. This emphasises the need for additional genomic sequencing of wild wheat relatives, so that donors of important genes can be identified more readily. However, even without donor sequence data, it is feasible to infer to some degree potential donor species simply based on the level of sequence homology.

A particularly important finding of this study was that numerous introgressions were found in single wheat accessions. Indeed, we identified 118 introgressions of 10 Mb or larger unique to single accessions in the collection. We are aware that the WHEALBI collection covers only a small fraction of the many thousands of wheat accessions that comprise the worldwide gene pool. Nevertheless, our data indicate that the wheat gene pool contains high numbers of rare but potentially valuable introgressions which must be first identified and then linked to agronomically important genes. We demonstrated the value of a specific introgression through QTL mapping, where we identified *PmPam*, a powdery mildew resistance locus originating from an introgression on the long arm of chromosome 7D found exclusively in the Turkish cultivar Pamukale. This major QTL segregates recessively and is the second recessive *Pm*-QTL reported on chromosome 7D, the other being *WTK4,* which is located on the short arm of chromosome 7D (Gaurav et al. 2021; Tang et al. 2023). The QTL identified in Pamukale confers resistance to multiple *Bgt* isolates originating from different geographical regions. This makes it a potentially valuable resource in resistance breeding. Moreover, the introgression containing *PmPam* is probably rare, as it is present in a single cultivar of the WHEALBI panel, and this might represent an additional advantage. In fact, broadly used, introgressed resistance genes are often overcome due to extended and strong selection pressure against avirulent pathogens (Kunz et al. 2023), which further emphasise the need of mapping new and rare resistance genes. Our finding illustrates that rare introgressions already present in the breeding pool can harbour unexploited, beneficial traits that can contribute to sustainable agriculture. This highlights the usefulness of introgression catalogues such as the one presented here.

### Inter-homoeologue recombination is frequent in the wheat gene pool

Our analysis of sequence coverage also revealed compelling evidence for recombination between homoeologous chromosomes in at least 54 accessions (Supp. Fig. 11, Supp. Table S6). Of these, at least eight do not seem to be independent, as they share one inter-homoeologue-recombination breakpoint, and all of them are either old cultivars or cultivars from Nepal (Supp. Fig. 12). Hexaploid wheat behaves as a genetic diploid since chromosomes of different subgenomes are assumed not to recombine. This bivalent behaviour is enforced by several genes among which the *Ph1* locus is the most important (Rey et al. 2017). It was suggested that inter-homoeologous recombination depends on the suppression of *Ph1* (Coombes et al. 2023). Our data suggest that despite this tight genetic control, inter-homoeologue recombination can occur in hexaploid wheat. While we identified most cases in landraces (14) and old cultivars (20), we also found evidence for such an event in the modern varieties such as Ritmo (Fig. 4a), Dekan, KWS Magic and Palesio (Supp. Fig. 13). Suppression of *Ph1* is used to enable chromosome pairing in introgression breeding (Martín et al. 2017). Therefore, some of the observed inter-homoeologous recombination events may be a by-product of introgression breeding. However, most of the events reported here were in old cultivars and landraces, which indicates that inter-homoeologue recombination also occurs naturally.

Previous studies showed that recombination between homoeologous chromosomes can occur. One study documented inter-homoeologue recombination in artificial tetraploid wheat (Zhang et al. 2020) and found recombination to occur primarily in low-copy sequences (i.e. genes). In cases where we could precisely identify the recombination breakpoints, we also found them inside orthologous genes, indicating that inter-homoeologue chromosome pairing occurs in syntenic positions. Similar findings were also made in rape seed and wheat using transcriptome data (He et al. 2017).

It must be emphasised that we identified these cases, because the inter-homoeologue recombination event was followed by unbalanced segregation, that resulted in recombinant segments being duplicated in a given genotype. A simple (symmetrical) exchange of chromosomal segments between homoeologues would not have been detectable purely based on sequence read coverage. It is therefore possible that the WHEALBI data set contains such symmetric exchanges between homoeologues which we were not able to detect.

**Fig. 7:**
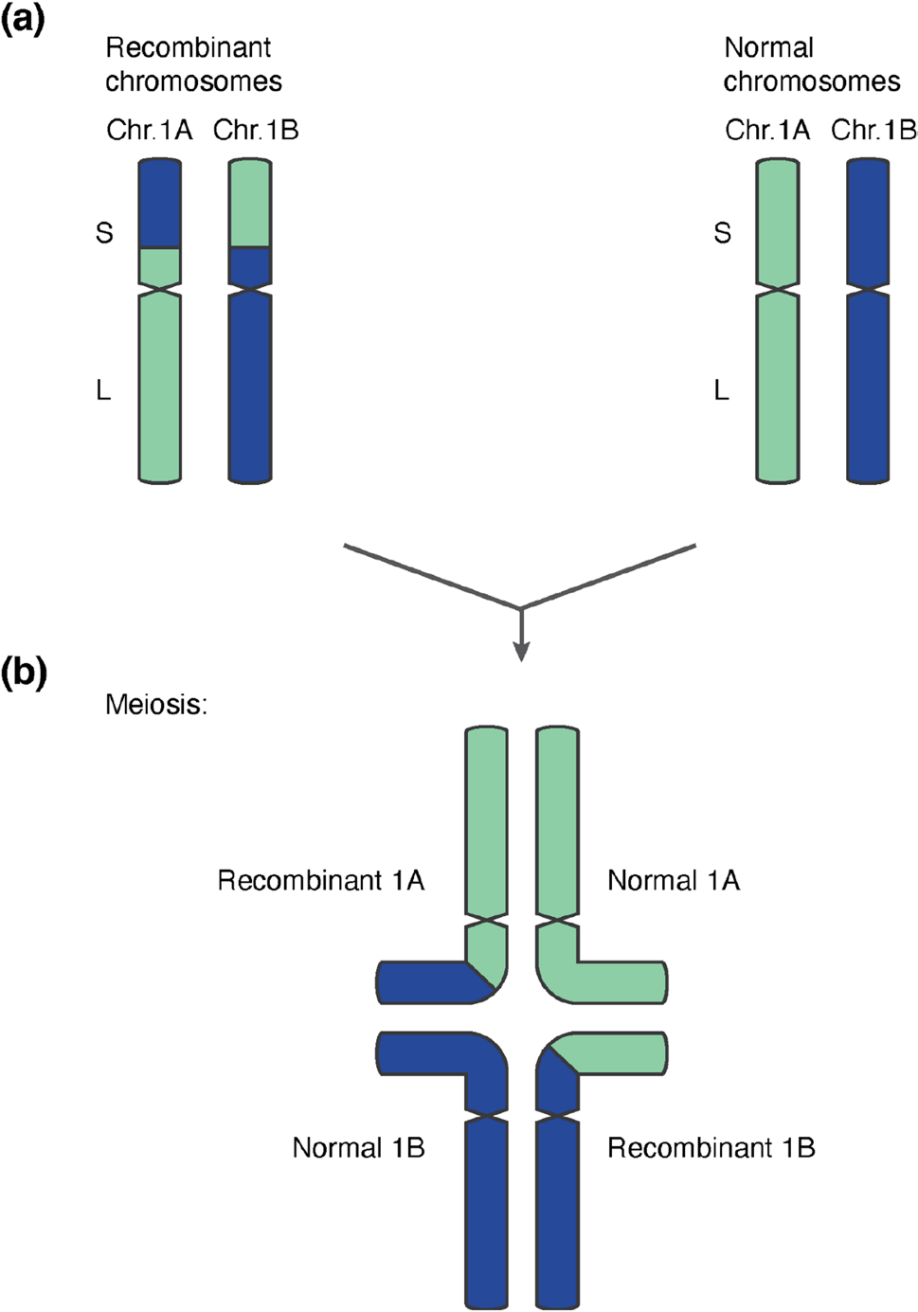
Schematic representation of cross-tetrad formation between inter-homoeologue recombinants and non-recombinants. **a** Schematic germline karyotype with one set of homoeologues carrying a balanced reciprocal translocation between homoeologues, and the other being normal. **b** Model for pairing of chromatids as cross-tetrads. The recombinant part of the short arm of Chr. 1A pairs with the homologous segment in the normal Chr. 1B. The non-recombinant parts of Chr. 1A pair normally. Commensurately, the recombinant part of the short arm of Chr. 1B pairs with the homologous segment in the normal Chr. 1A, while the non-recombinant parts of Chr. 1B pair normally

In this context it is interesting that we also found cases that presumably resulted from multiple, sequential inter-homoeologue recombination events (Fig. 5). Such events depend on an initial, symmetric, inter-homoeologue recombinant in a homozygous genotype. Further recombination and duplication events led to the observed types (Fig. 5). This would mean that symmetric recombinants must be able to recombine with non-recombinant wheat lines, or they would likely be selected against (either naturally or by humans) and rapidly decline in frequency. Indeed, maintenance of heavily re-arranged chromosomes in the wheat gene pool has been reported previously: for example, the wheat variety Arina, like other European winter wheat lines (Walkowiak et al. 2020), has a major rearrangement where the short arms of chromosomes 5B and 7B are exchanged, but the recombinant Arina can be crossed without restrictions with non-recombinant wheat lines (Walkowiak et al. 2020; Kolodziej et al. 2021). The underlying genetic mechanisms are well known: recombinant chromosomes would recombine with non-recombinant ones by forming cross-tetrads, a constellation that allows homologous chromosome segments to pair correctly (Copenhaver et al. 2000; Golczyk et al. 2008). A schematic description of the proposed mechanism is shown in Fig. 7.

In conclusion, our study confirms that the wheat gene pool comprises a plethora of accessions with complex patterns of chromosomal introgressions and chromosomal rearrangements and further provides an introgression landscape easily accessible at https://github.com/matthias-heuberger/whealbi_introgression_viewer, providing novel tools for future breeding improvement.

## Supplementary Information

Below is the link to the electronic supplementary material.

Supplementary file1 (PDF 14146 KB)

Supplementary file2 (XLSX 14151 KB)

## Data Availability

Linkage map, genotype and phenotype information of the Pamukale x Frisal F2 progeny are available in Supp. Tables S5 and S4, respectively. Normalised coverage data and introgression assignment are available at: https://zenodo.org/records/10406469. Genome assemblies are publicly available and available for download in the following locations. IWGSC RefSeq v2.1: https://urgi.versailles.inra.fr/download/iwgsc/IWGSC_RefSeq_Assemblies/v2.1/; ArinaLrFor, Claire, Jagger, Julius, Kariega, Lancer, Landmark, Mace, Renan, Stanley, SYMattis: https://plants.ensembl.org/info/data/ftp/index.html; Attraktion: https://www.ebi.ac.uk/ena/browser/view/GCA_918797515.1. The WHEALBI exome capture data are publicly available and can be obtained in NCBI with the BioProject number PRJNA524104. The genomic sequence reads of wheat relatives are publicly available in the following locations: *T. timopheevii*, *Ae. ventricosa*, *T. ponticum*—NCBI BioProject number PRJNA544491, *Ae. markgrafii* and *Ae. speltoides*—ENA ID PRJEB49121, *T. monococcum—*ENA ID PRJEB61155. GBS data are publicly available in ENA under the ID PRJEB41976. An R shiny application that can be used to visualise the coverage ratio data generated for this manuscript is available at: https://github.com/matthias-heuberger/whealbi_introgression_viewer.
