# Supplementary material for: Analysis of a global wheat panel reveals a highly diverse introgression landscape and provides evidence for inter-homoeologue chromosomal recombination
Source: Theor Appl Genet. 2024 Sep 28;137(10):236. doi: 10.1007/s00122-024-04721-x (PMC11438656; doi:10.1007/s00122-024-04721-x)
Supplement: Supplementary file 1 — Supplementary file1 (PDF 14146 KB) [file 122_2024_4721_MOESM1_ESM.pdf]

Supplementary Figures

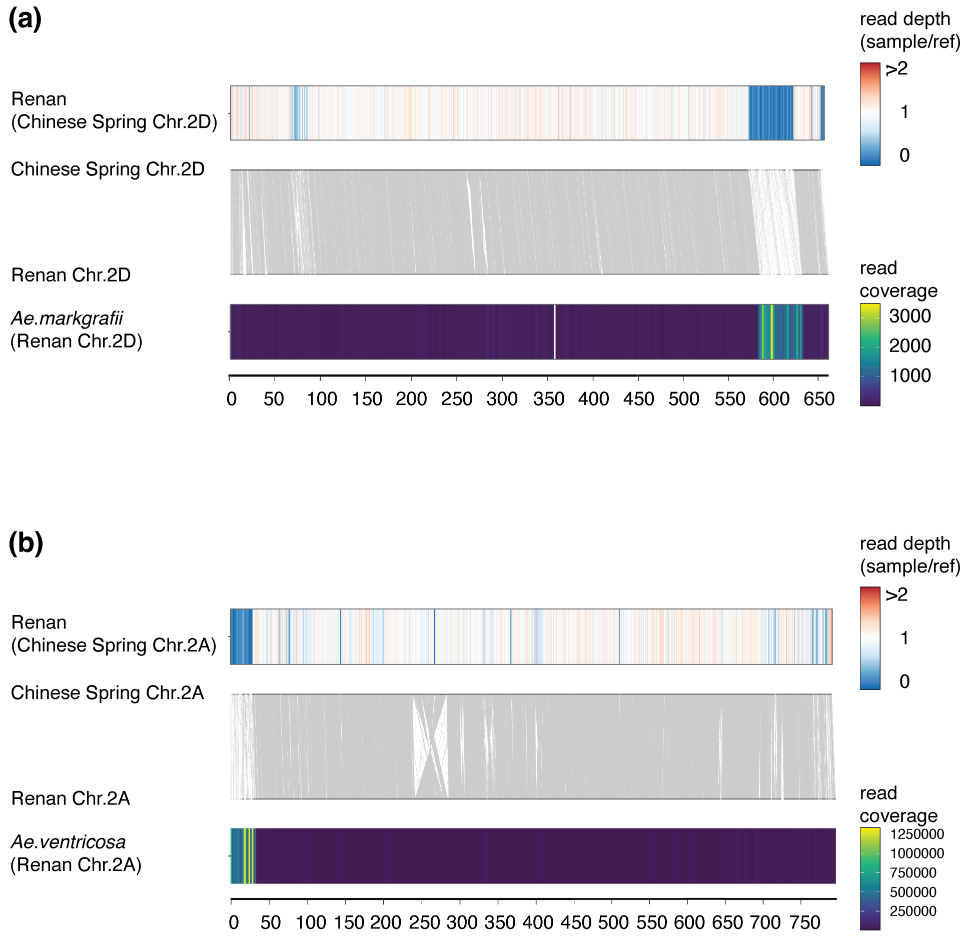

Supp. Fig. 1. Confirmation of previously described introgressions in Renan **(a)** The top drawing shows the coverage of normalised exome capture reads of Renan on Chr. 2D. The middle drawing shows the chromosome collinearity between Chinese Spring Chr. 2D and Renan Chr. 2D. At the bottom, the read coverage of *Ae. markgrafii* WGS reads mapped on Renan Chr. 2D is given. Values for exome capture were calculated in 500 kb bins and WGS in 2 Mb bins. **(b)** The top drawing shows the coverage of normalised exome capture reads of Renan on Chr. 2A. In the middle, the chromosome collinearity between Chinese Spring Chr. 2A and Renan Chr. 2A is shown and at the bottom, the read coverage of *A. ventricosa* WGS reads mapped on Renan Chr. 2D given. Values for exome capture were calculated in 500 kb bins and WGS in 2 Mb bins.

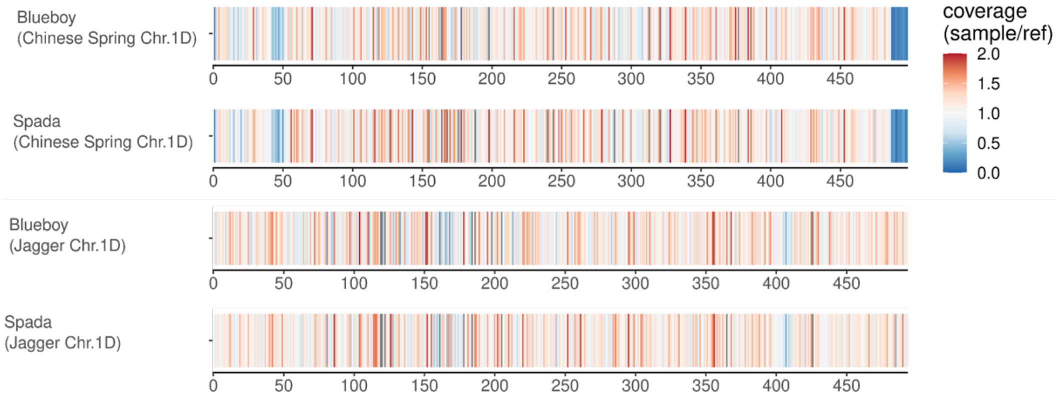

Supp. Fig. 2. Blueboy and Spada contain the same introgression as Jagger. GBS data of Blueboy and Spada mapped on Chinese Spring Chr. 1D and Jagger Chr. 1D. At the distal end of Chinese Spring Chr. 1D Blueboy and Spada show low coverage, while they show normal coverage at the distal end Chr. 1D of Jagger. Values were calculated in 2 Mb bins and normalised by GBS read coverage of the respective reference genotype against which the reads were mapped.

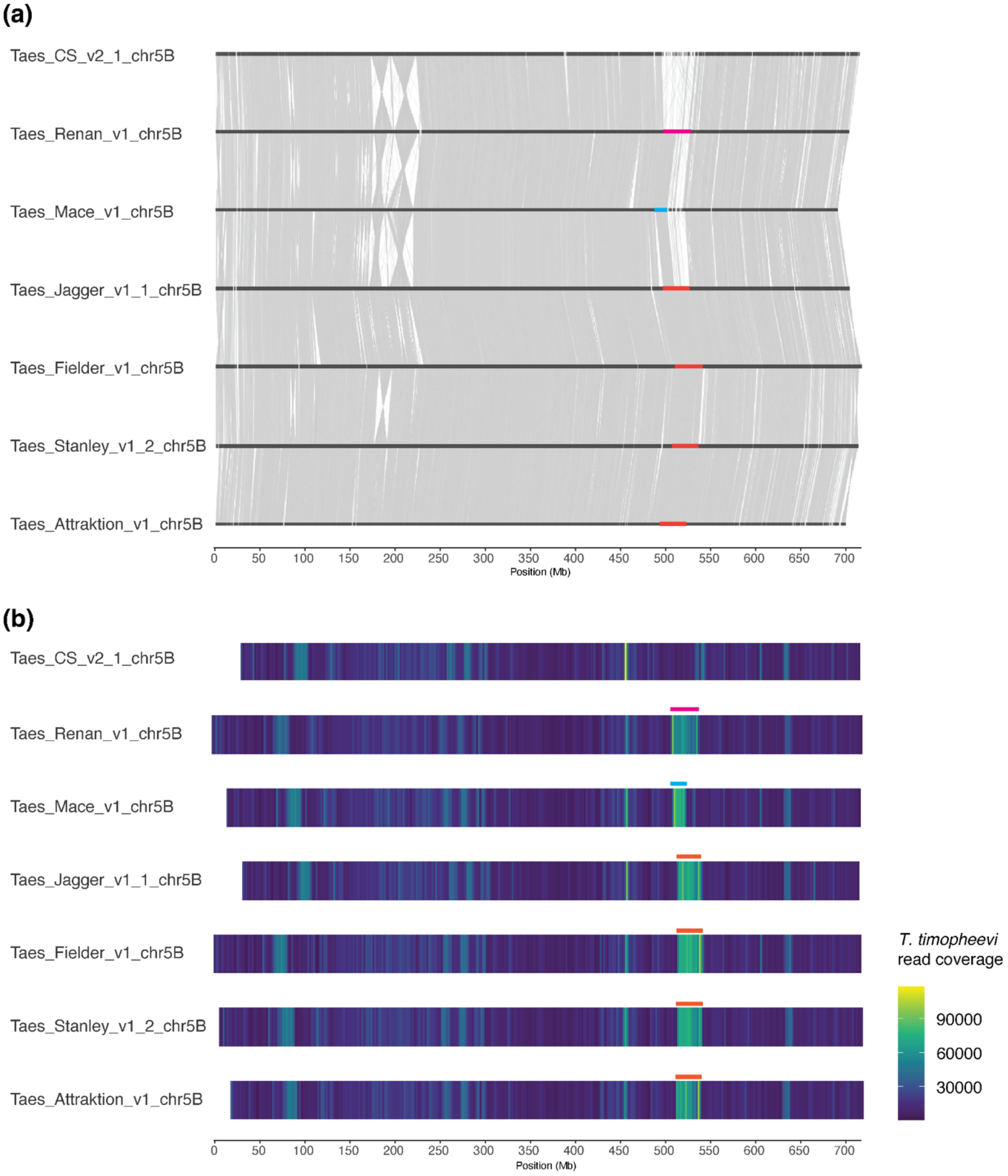

Supp. Fig. 3. Reference-quality genome assemblies (RQAs) have three different versions of the 5B introgression. **(a)** Chromosome collinearity for Chr. 5B between Chinese Spring and six RQAs containing the 5B introgression. Chromosomes were arranged according to similarity in the region of the introgression. **(b)** *T. timopheevii* WGS heat map showing read coverage of reads mapped on Chr. 5B of Chinese Spring and six RQAs containing the 5B introgression. Values were calculated in 2 Mb bins. To allow visual comparison of the introgression region, the chromosomes were aligned based on collinearity just before the introgression. Scale at the bottom is aligned to Chr. 5B of Fielder.

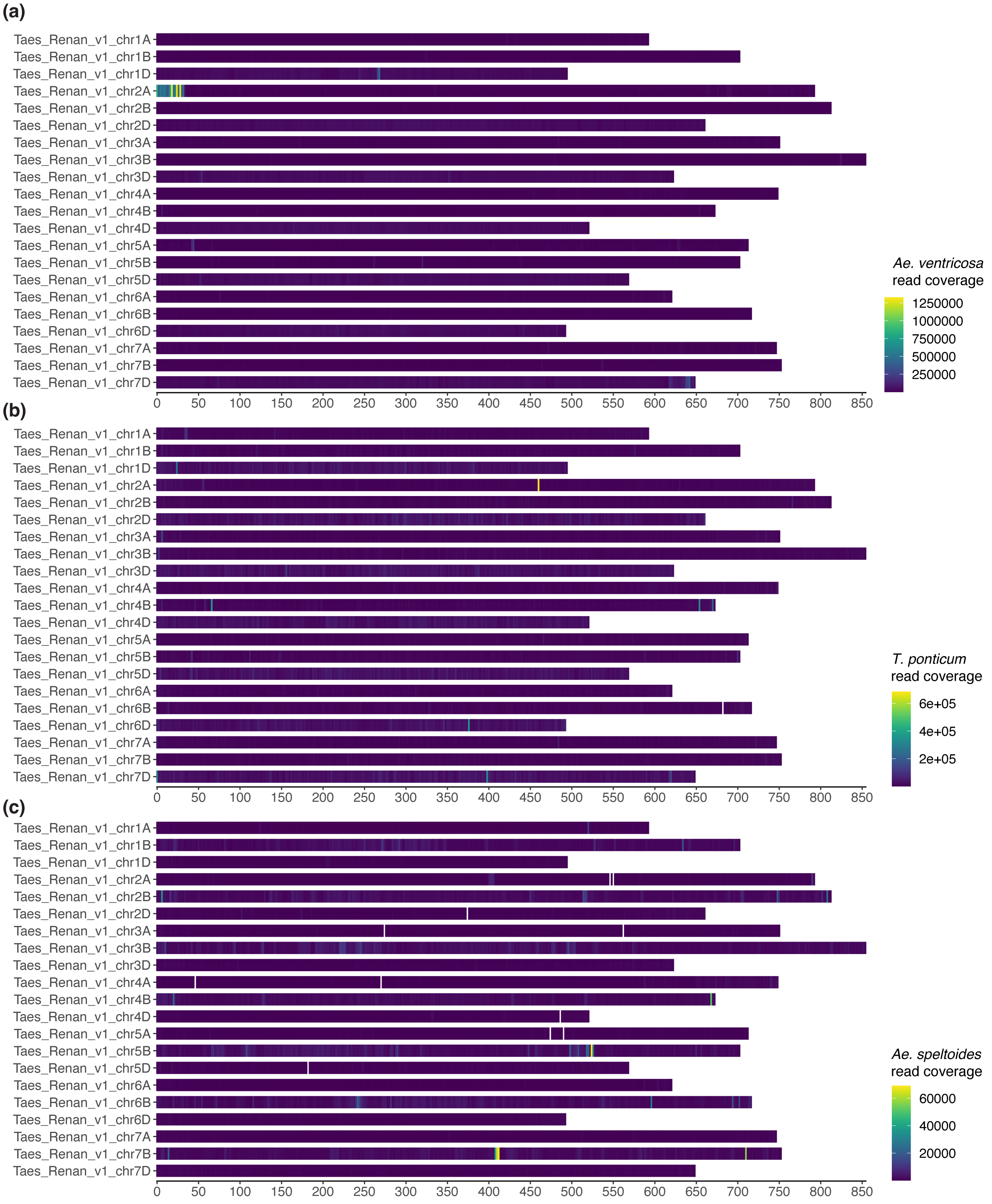

Supp. Fig. 4. Mapping of wild wheat relatives onto the Renan genome assembly **(a)** Read coverage of WGS reads from *Ae. ventricosa* mapped on Renan. **(b)** Read coverage of WGS reads from *T. ponticum* mapped on Renan. **(c)** Read coverage of WGS reads from *Ae. speltoides* mapped on Renan. Values were calculated in 2 Mb bins.

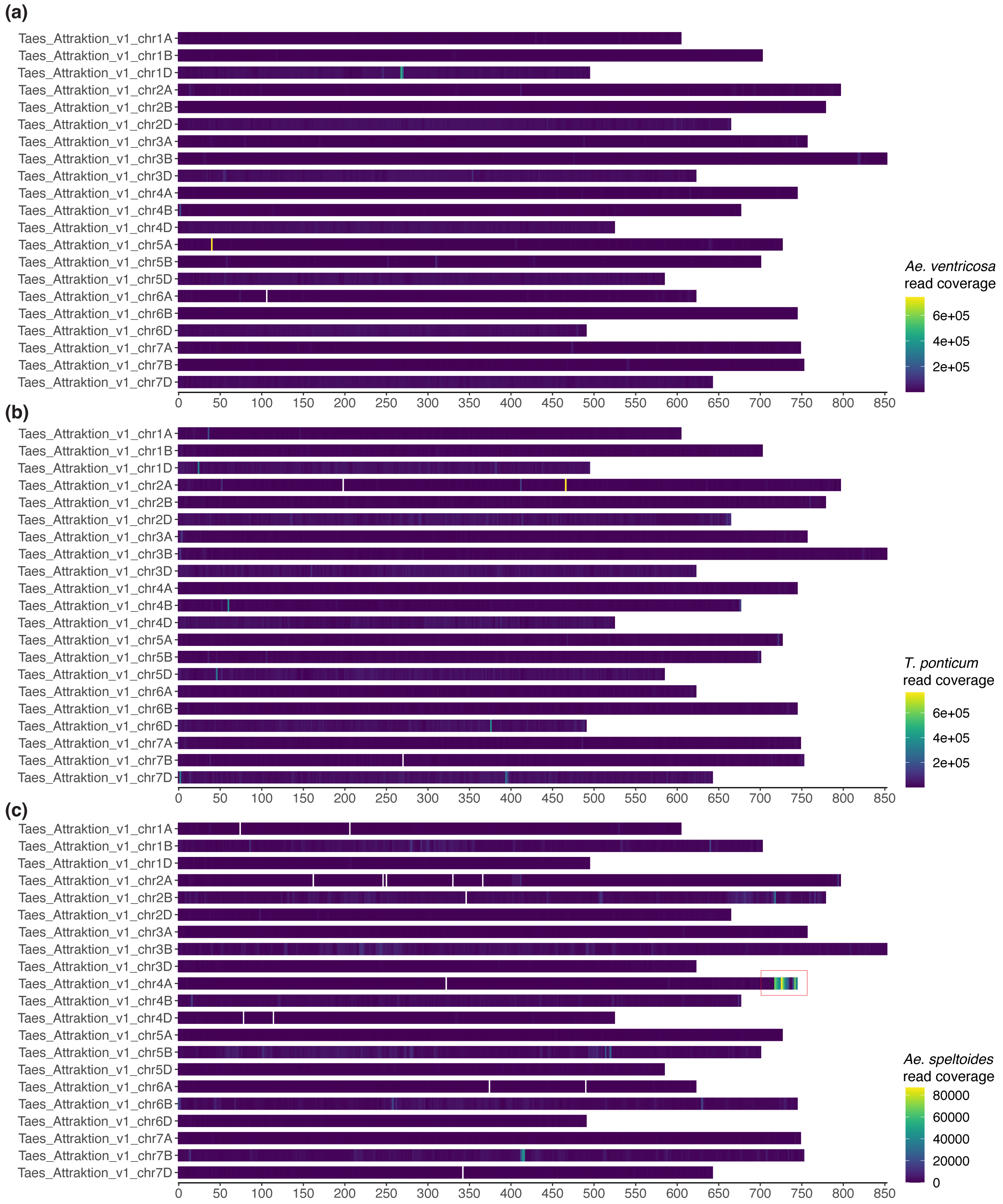

Supp. Fig. 5. Mapping of wild wheat relatives onto the Attraktion genome assembly **(a)** Read coverage of WGS reads from *Ae. ventricosa* mapped on Attraktion. **(b)** Read coverage of WGS reads from *T. ponticum* mapped on Attraktion. **(c)** Read coverage of WGS reads from *Ae. speltoides* mapped on Attraktion. Known *Ae. speltoides* introgression (Keilwagen et al. 2022) highlighted by red rectangle. Values were calculated in 2 Mb bins.

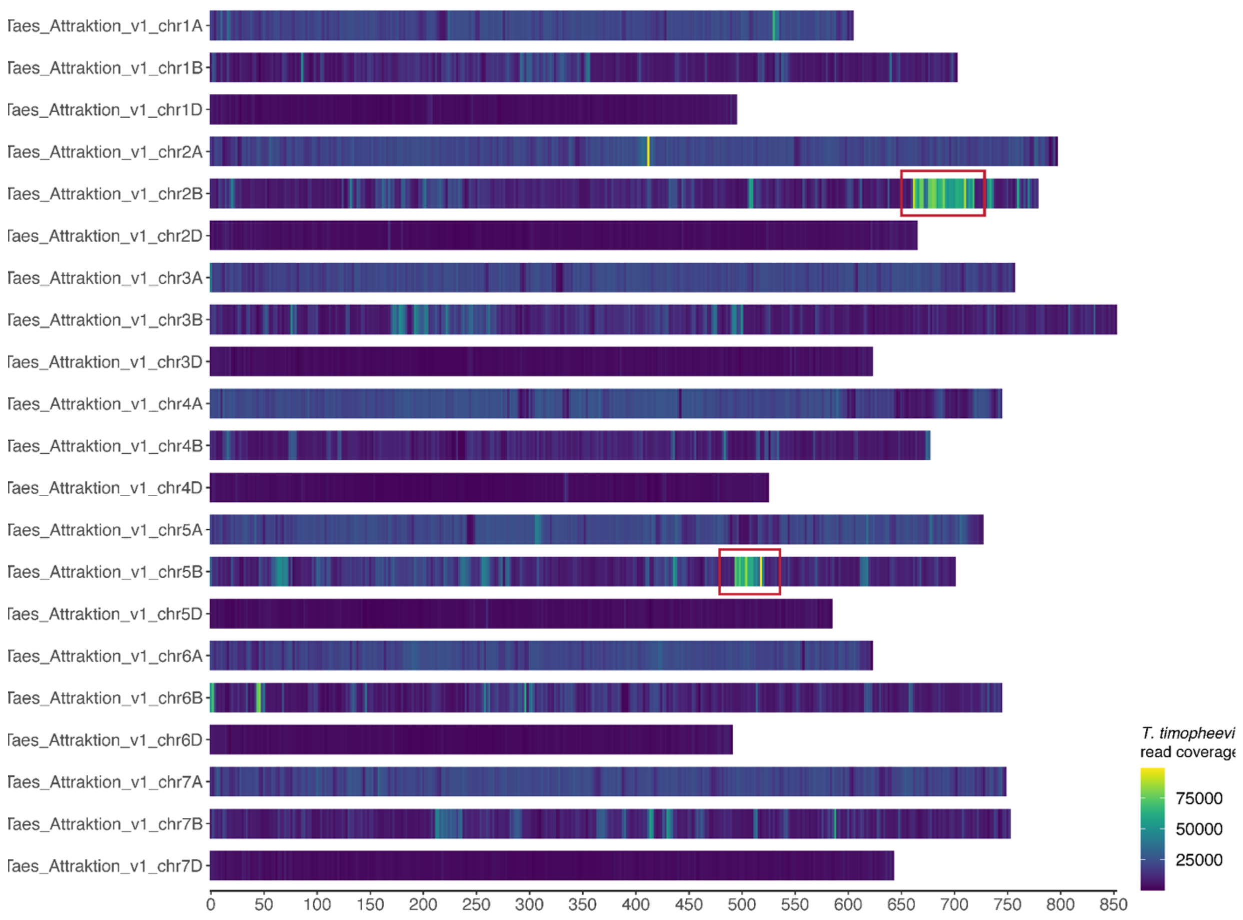

Supp. Fig. 6. Mapping of wild *T. timopheevii* onto the Attraktion genome assembly. Heatmap showing the read coverage of *T. timopheevii* WGS reads mapped on the genome assembly of Attraktion. The known *T. timopheevii* introgression on Chr. 2B and the introgression on Chr. 5B are highlighted by a red rectangle. Values were calculated in 2 Mb bins.

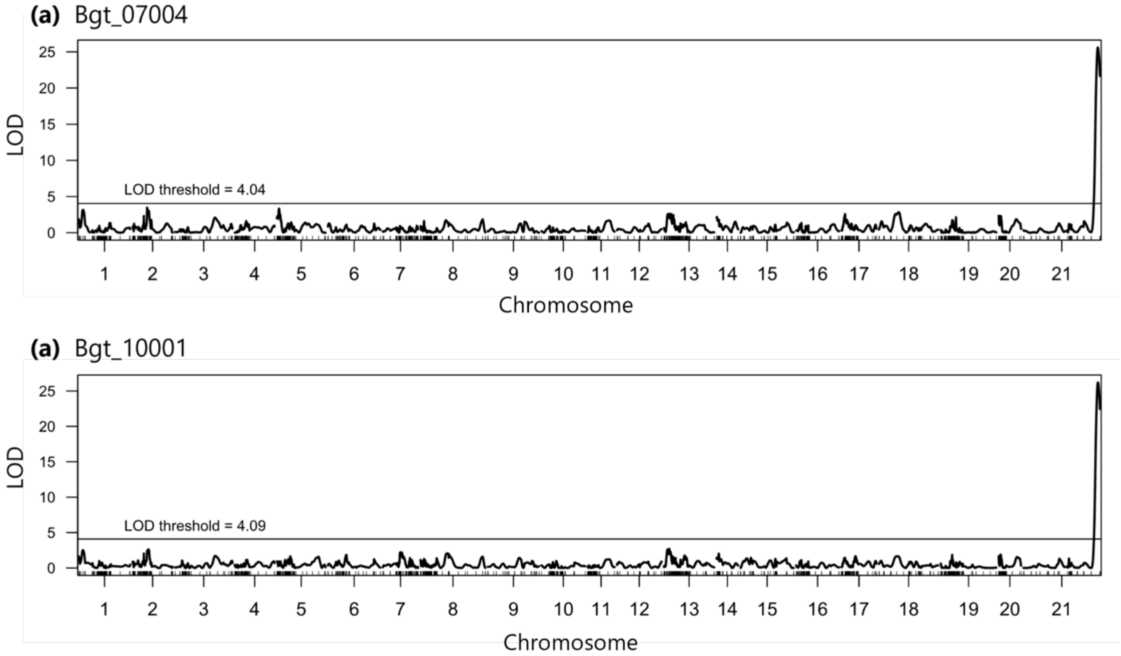

Supp. Fig. 7. Identification of a powdery mildew resistance locus in chromosome 7D (chromosome 21 in the figure). The powdery mildew isolates **(a)** Bgt_07004 and **(b)** Bgt_10001 reveal the same QTL. The LOD thresholds are indicated in the graphs and were calculated with the *qtl* package in R with 500 permutations.

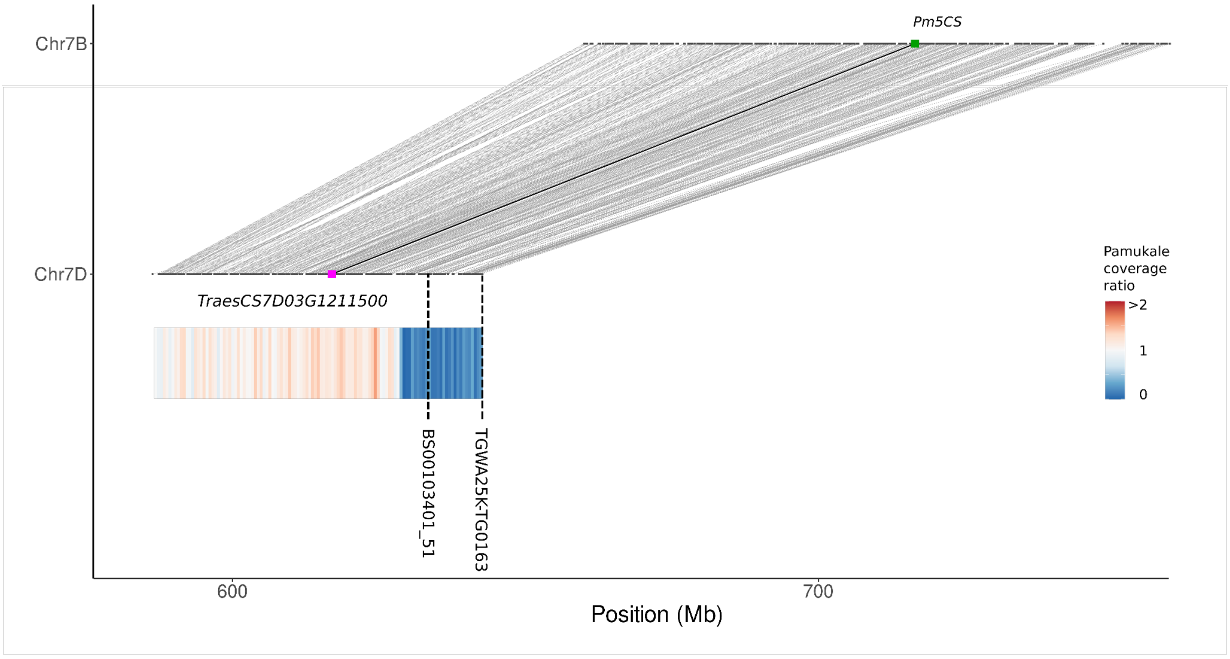

Supp. Fig. 8. Synteny analysis between the ends of Chinese Spring chromosomes 7B and 7D. Synteny was determined based on blastn queries of the respective gene coding sequences against each other. Pm5CS and its 7D homoeologue are indicated with a green and a pink dot respectively. The position of two SNP markers associated with the *PmPam* QTL is indicated by dashed lines.

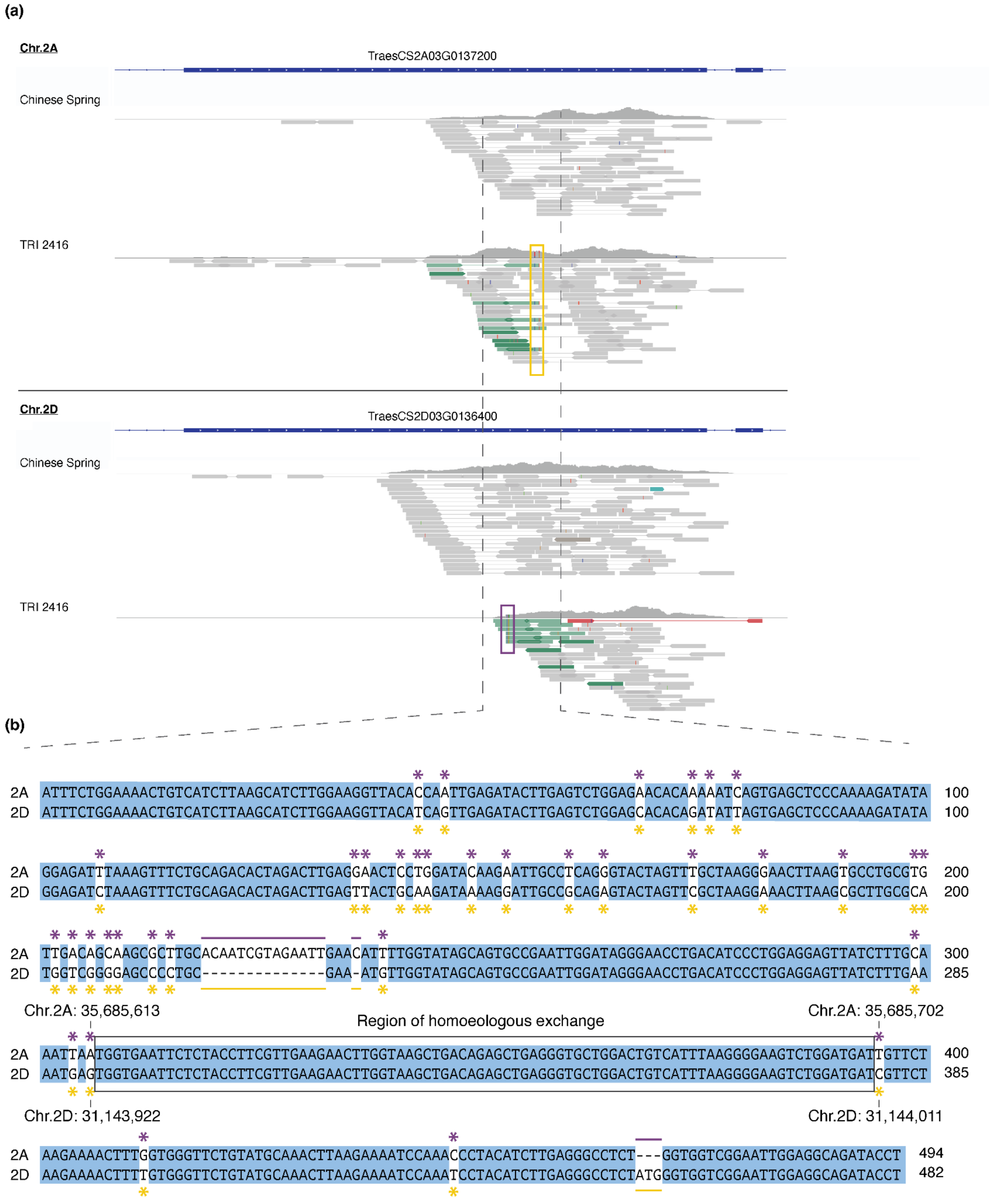

Supp. Fig. 9. Identification of inter-homoeologue recombination breakpoint. **(a)** Visualisation of mapped reads from Chinese Spring and TRI 2416 exported from IGV. Mate-pairs going across the breakpoint (one mate mapping to Chr.2A the other to Chr.2D) were coloured in green. Reads that show both 2A- and 2D-specific SNPs in the same read (i.e. chimeric reads) and framed by coloured rectangles. **(b)** Alignment of Chr.2A and Chr.2D in the region surrounding the suspected breakpoint 2A- or 2D-specific SNPs are indicated by asterisk and specific InDels are indicated by coloured lines. There were no reads on either 2A or 2D in this segment, where the corresponding mate mapped to the syntenic position in 2B.

(a)

(b)

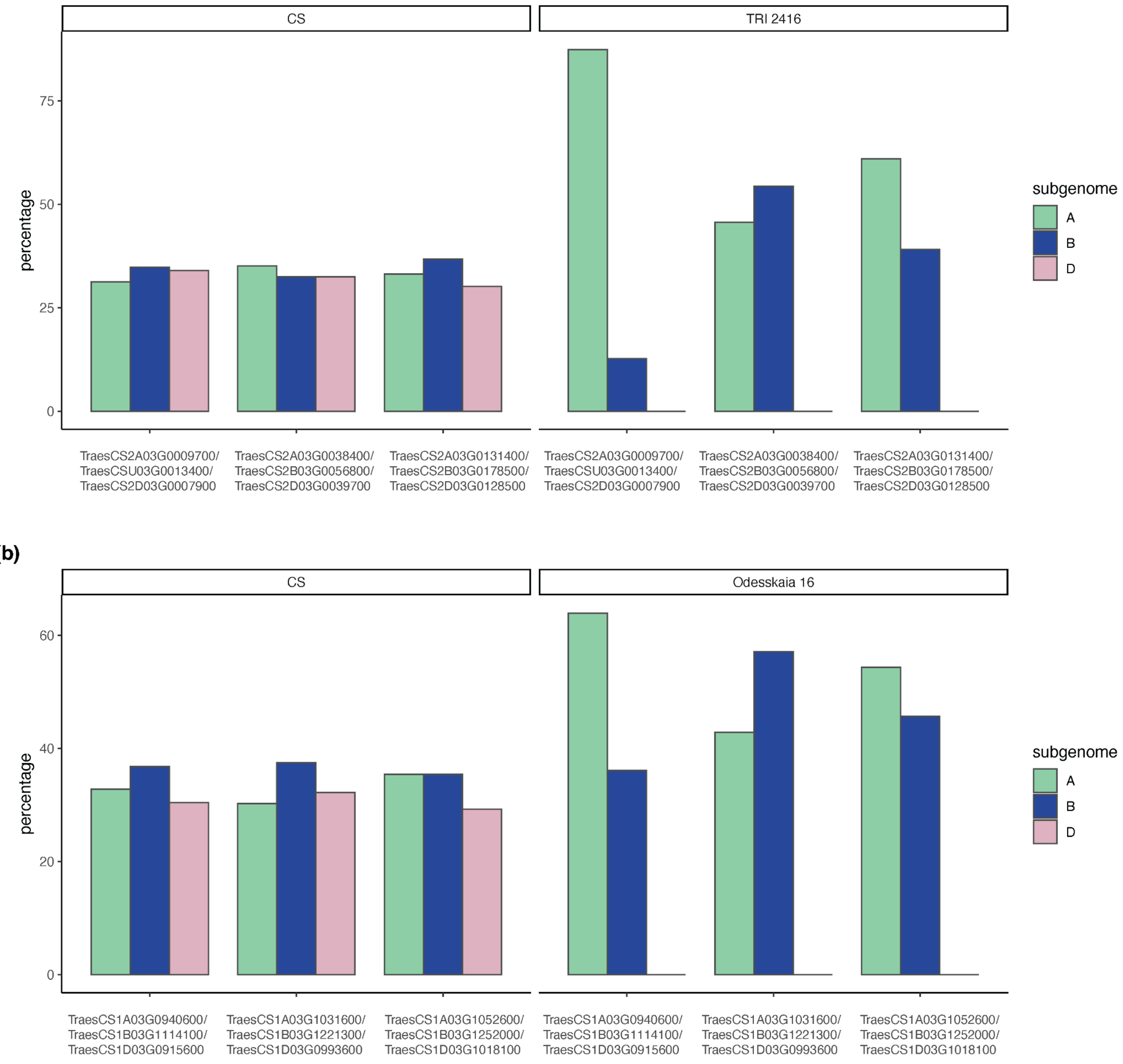

Supp. Fig. 10. Amplicon-sequencing results of homoeologous gene triads **(a)** Triads for HE event involving Chr2A and Chr2D in TRI 2416. **(b)** Triads for HE-event involving Chr1A and Chr1D in Odesskaia 16.

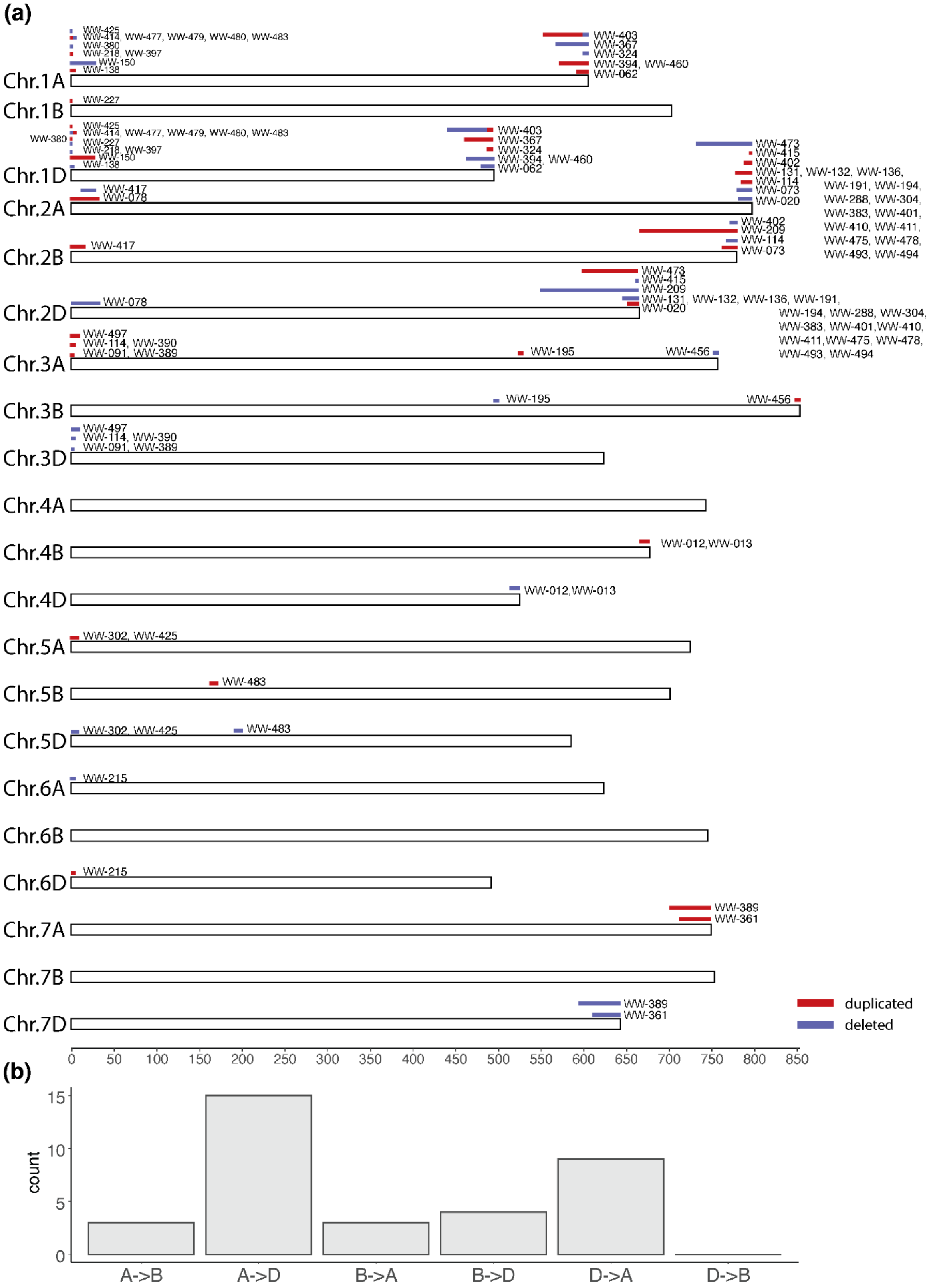

Supp. Fig. 11. Overview of inter-homoeologue recombination events **(a)** Duplicated segments are marked with a red line and deleted segments are marked by a blue line. The accessions carrying the events are specified. In case an event is likely shared between accessions (i.e. same origin, based on border and origin of sample) only one line is shown for this event and the names are grouped. (b) Frequencies of inter-homoeologue recombination events separated for the different subgenome origins.

For example, the designation A->B means that an A subgenome segment is duplicated and the syntenic B genome segment is missing.

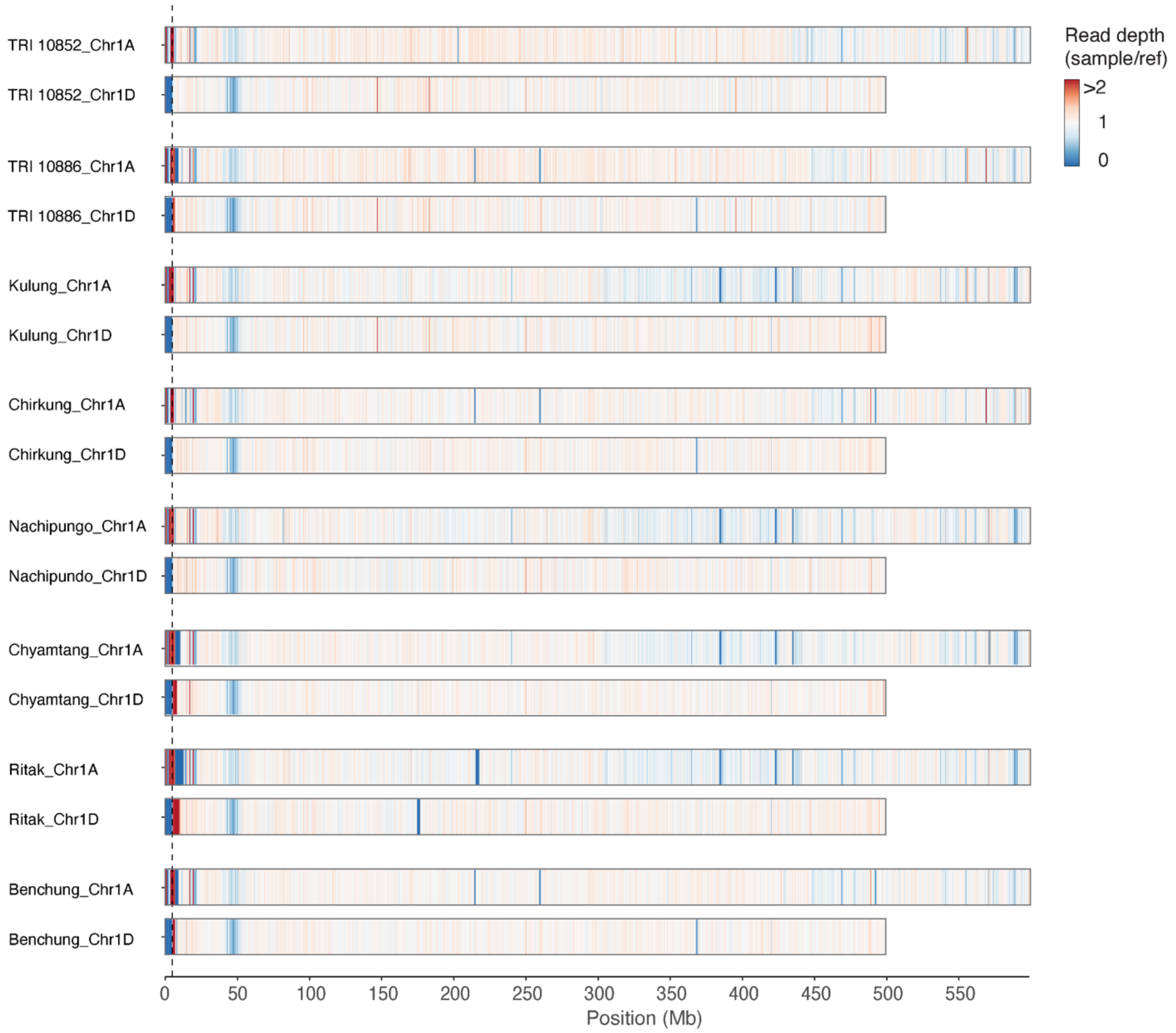

Supp. Fig. 12. Suspected inter-homoeologue recombination event common to eight Nepalese accessions. Exome capture reads were mapped to the genome of Chinese Spring and coverage ratio was calculated in 500 kb bins. The 8 shown accessions all share one inter-homoeologue recombination event between chromosome 1A and 1D. The location of the breakpoint in chromosome 1D is indicated by a dashed line.

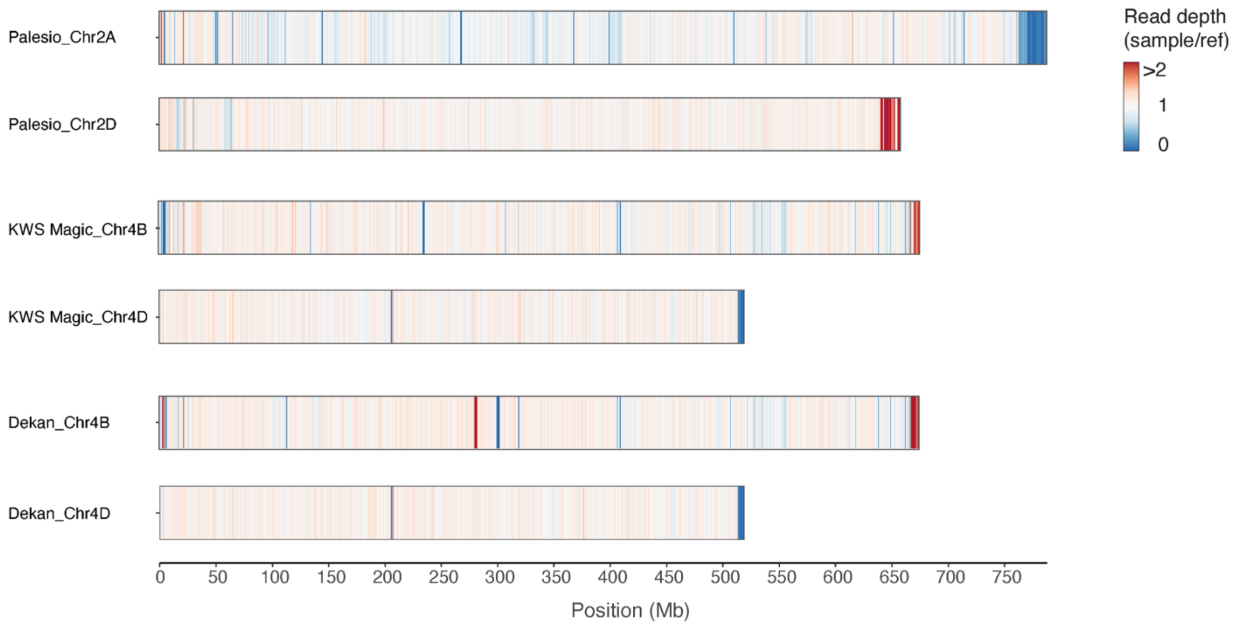

Supp. Fig. 13. Suspected inter-homoeologue recombination events in current varieties. Exome capture reads were mapped to the genome of Chinese Spring and coverage ratio was calculated in 500 kb bins.
